# SANA: cross-species prediction of Gene Ontology GO annotations via topological network alignment

**DOI:** 10.1038/s41540-022-00232-x

**Published:** 2022-07-20

**Authors:** Siyue Wang, Giles R. S. Atkinson, Wayne B. Hayes

**Affiliations:** Department of Computer Science, University of California, Irvine, CA 92697-3435 USA

**Keywords:** Dynamic networks, Protein design, Biochemical networks, Molecular biology, Genetic interaction

## Abstract

Topological network alignment aims to align two networks node-wise in order to maximize the observed common connection (edge) topology between them. The topological alignment of two protein–protein interaction (PPI) networks should thus expose protein pairs with similar interaction partners allowing, for example, the prediction of common Gene Ontology (GO) terms. Unfortunately, no network alignment algorithm based on topology alone has been able to achieve this aim, though those that include sequence similarity have seen some success. We argue that this failure of topology alone is due to the sparsity and incompleteness of the PPI network data of almost all species, which provides the network topology with a small signal-to-noise ratio that is effectively swamped when sequence information is added to the mix. Here we show that the weak signal can be detected using multiple stochastic samples of “good” topological network alignments, which allows us to observe regions of the two networks that are robustly aligned across multiple samples. The resulting network alignment frequency (NAF) strongly correlates with GO-based Resnik semantic similarity and enables the first successful cross-species predictions of GO terms based on topology-only network alignments. Our best predictions have an AUPR of about 0.4, which is competitive with state-of-the-art algorithms, even when there is no observable sequence similarity and no known homology relationship. While our results provide only a “proof of concept” on existing network data, we hypothesize that predicting GO terms from topology-only network alignments will become increasingly practical as the volume and quality of PPI network data increase.

## Introduction and motivation

While much effort is devoted to prediction of protein function by mapping sequence and structure to function, not all proteins have analogs to ones with known function, and the sequence-function relationship is far from 1-to-1: there can be functional similarity in the absence of sequence similarity^1–3^, and conversely identical sequences can possess multiple, completely different functions^3–5^. Confusing matters further, minor changes in sequence can result in significant changes to function^6,7^, and similar structure does not always imply similar function^8^. However, one thing is Fcertain: a protein’s function is intimately tied to its set of interaction partners. Since protein–protein interaction (PPI) networks can be measured directly, they potentially provide a road map to function that avoids the complexities of relating sequence and structure to function.

Given that all life on Earth is related, and that proteins derived from genes that have even a remote a common ancestor often share not only sequence but also functional similarity^9^, it is reasonable to hypothesize that proteins in different species that share common function might be aligned together by a network alignment driven to maximize the number of common interactions observed in an alignment. Stated in terms of graph theory, we expect that nodes in two different PPI networks that share common function should also share similar topology among their network interactions. More succinctly, we expect network topology and protein function to be related. Importantly, the statement that proteins with similar function are likely to share similar interaction partners does not require any sequence relationship between the proteins claimed to have similar function; similar network connectivity may be sufficient. This is the basis on which we can hypothesize that topological network alignment may be able to discover inter-species functional orthology even in the absence of sequence similarity.

Unfortunately, PPI networks for most species are noisy^10^, incomplete^11^, and biased^12,13^. Such data make it difficult to detect common network topology, so that “failure to find network conservation [between] species [is] likely due to low network coverage, not evolutionary divergence”^14^. For example, the most recent human PPI network from BioGRID (version 3.5.184, released April 2020) contains 368,005 unique interactions amongst 17,815 unique human proteins; for comparison, the next most complete mammal in the same release is mouse, which contains barely 6% of the interactions of human, at only 22,903 interactions amongst 7543 unique mouse proteins. (Note that the numbers given on the BioGRID website for each species include interactions with proteins outside the named species. These must be removed in order to extract the PPI network of the desired species. We also remove self-interactions, to simplify the graph theory.) Given that the number of edges in the human BioGRID network has consistently grown by about 30% each year for the past decade and shows no signs of leveling off, both networks must be considered incomplete.

Given the highly disparate levels of PPI network completeness between species, it may come as no surprise that, among the more than fifty attempts in the literature at aligning PPI networks, very few have been able to demonstrate a statistically significant relationship between topological and functional or semantic similarity, with most successes involving local network topology as described by *graphlets*^15–23^. Instead, most authors understandably augment the objective function for *network* alignments with *sequence* similarity of aligned proteins, and such methods met with early success^24^ and continue to meet with success. The problem with this approach is one of signal to noise: any novel functional information hidden in the weak signal that may exist in the common topology between today’s (highly incomplete) networks is likely to be “drowned out” by the much stronger—and already well-understood—signal that exists between proteins of similar sequence. Thus, *network* alignments driven by an objective function that includes *sequence* similarity may lose the opportunity to learn from any weak signal that may exist in the topology of PPI networks but is obscured by little or no sequence similarity.

What has been lacking in topology-driven network alignments to date is a way to cut through the noise and incompleteness of existing PPI network data to find the functional information hidden in the noisy and incomplete topological data. Our solution is to “fight fire with fire”, and utilize intentionally generated randomness to separate signal from noise. Given two networks whose topological similarity we wish to explore, we randomly walk through the alignment search space, eventually converging on a network alignment that exposes a near-optimal amount of topological similarity. Since each random walk takes a different path towards optimality, nodes that share the greatest amount of topological similarity have the greatest chance of becoming aligned across independent paths taken towards a near-optimal solution. Our random walk through search space is generated using simulated annealing, which has a rich history of success in optimizing NP-complete problems^25–37^. Its randomness is key: each run of our Simulated Annealing Network Aligner, or SANA^38,39^, follows a different, randomized path towards an alignment that uncovers close to the maximum amount of common topology that can be discovered between two networks^40^. Since each path to a near-optimal alignment is different, each run of SANA produces a different alignment-but all alignments have nearly the same, close-to-optimal score. SANA effectively produces a random sample from the frontier of near-optimal alignments. If there is any weak signal of true common topology between a pair of PPI networks, we would expect such common topology to re-appear across these independently generated, near-optimal alignments with a frequency above random. In other words, the alignment of truly similar regions is repeatable. For example, if SANA independently generates 100 alignments, the better-than-random re-alignment of regions with better-than-random topological similarity manifests as a better-than-random chance that individual pairs of proteins embedded in these regions will appear at frequencies that are higher than random chance would allow. Those pairs of proteins that appear most frequently will tend to lie in regions with the greatest amount of topological similarity, and consequently we would expect such aligned pairs of proteins to have the highest functional similarity among our aligned protein pairs.

We dub the result Network Alignment Frequency, or NAF. The NAF of a pair of proteins (*p*, *q*) from different species measures the propensity that they will align repeatedly across multiple independently generated near-optimal alignments. We find that NAF strongly correlates with Resnik’s Semantic Similarity (cf. Fig. 2).

### Contribution

In this paper, our network alignments are driven by network topology alone: the only input is two lists of protein–protein interactions (PPIs)—one PPI network for each species. We demonstrate that SANA’s network alignment frequency (NAF) not only correlates with Resnik similarity, but is able to predict novel GO annotations, even in the absence of detectable sequence similarity. Our results are validated in two ways: with predictions made in 2010 validated en masse by comparison with GO terms available in 2020 (10 years later); and on a smaller scale, with predictions made using data available in later 2018 manually validated by literature search today. The latter predictions, based on high NAF scores, were made by transferring GO annotations from a mouse protein that was annotated with GO terms, to a human protein that lacked such annotations and had no detectable sequence similarity according to NCBI PSI-BLAST, nor any known homology relationship using the latest available orthology databases (see “Methods”).

Finally, we note that it is not merely the increase in data volume over the past decade, but our method that has enabled these results, since our 2010-based predictions used *only* data that was available as of April 2010, and none of the network alignment algorithms published in the intervening decade has successfully leveraged topology alone to predict a significant number of GO annotations with acceptable accuracy.

**Fig. 1:**
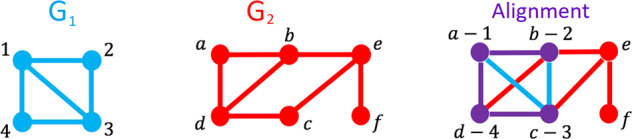
A schematic depiction of a 1-to-1 Pairwise Global Network Alignment (PGNA). The input graphs are *G*_1_ (blue, with fewer nodes), and *G*_2_ (red). The network alignment can be depicted itself as a network with two types of nodes (purple and red) and three types of edges (purple, blue, and red). Aligned nodes and edges are purple, depicting a mix of red and blue. Unaligned nodes and edges retain the color of the graph they came from. Note that in the aligned network, two common measures of topological network similarity can easily be interpreted visually: EC = ∣purple edges∣/∣purple+blue edges∣, while *S*^3^ = ∣purple edges∣/∣edges of all colors between purple nodes∣.

**Fig. 2:**
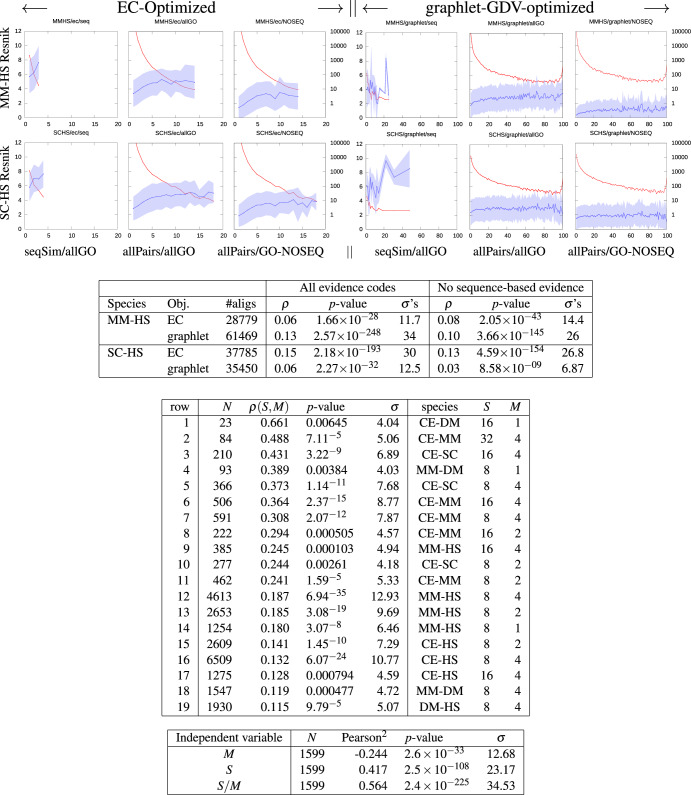
Two rows of figures at top plot the Resnik-based similarity vs. NAF between mouse-human (top row) and yeast-human (bottom row). Top Table: Pearson correlation (*ρ*) and statistical significance of the plots. Middle Table: Filtering for well-annotated proteins, we see higher Pearson correlations between NAF and Resnik score (allowing all evidence codes) that result when filtering for well-annotated protein pairs in EC-driven alignments; *N* is the number of aligned protein pairs for which *both* proteins are annotated with at least *S* GO terms that are each annotate at most *M* proteins per species. We exhaustively list every pair of BioGRID species for which the Pearson *p*-value is <10^−2^ for *S* ≥ 8 and *M* ≤ 4; the table is sorted by *ρ*(*S*, *M*). Bottom Table: Pearson correlation between *M*, *S*, and *ρ*(*S*, *M*) above, across all species and values *M* and *S* for which *ρ*(*S*, *M*) was statistically significant (see text for further discussion).

**Fig. 3:**
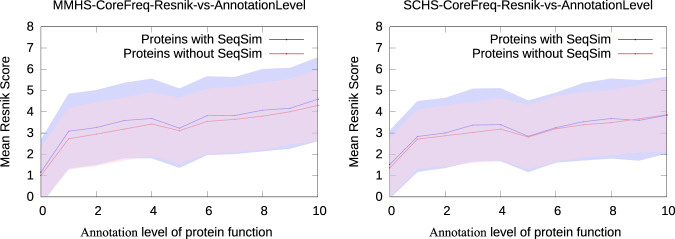
Protein pairs aligned by network topology alone have equal Resnik similarity—not including sequence-based evidence—independent of whether they possess sequence similarity. Note the horizontal axis here is no longer NAF, it is annotation level of aligned pairs across all those with NAF 2% or higher. We plot mean Resnik score as a function of annotation level for MMHS (left) and SCHS (right). In each plot, aligned protein pairs (*p*, *q*) are binned along the horizontal axis into the integer part of the NetGO-based annotation detail^111^ of the lesser understood protein. The vertical axis is mean Resnik score, with shading out to 1*σ* standard deviation of the pairs in that bin. Blue is protein pairs with sequence similarity, red is those without. In all cases, the Pearson correlations are above 0.35 and have *p*-values below 10^−300^ before binning to take the mean, while the *p*-value of Pearson correlation of the binned means are about 2 × 10^−3^; the difference between the means has *p*-value above 0.4—i.e., far from statistical significance. The Resnik scores here are significantly lower than those in Fig. 2 for two reasons: first, we have, of necessity, removed all sequence-based evidence, and second, the mean is dominated by the high number of low-NAF (NAF = 2%) pairs.

**Fig. 4:**
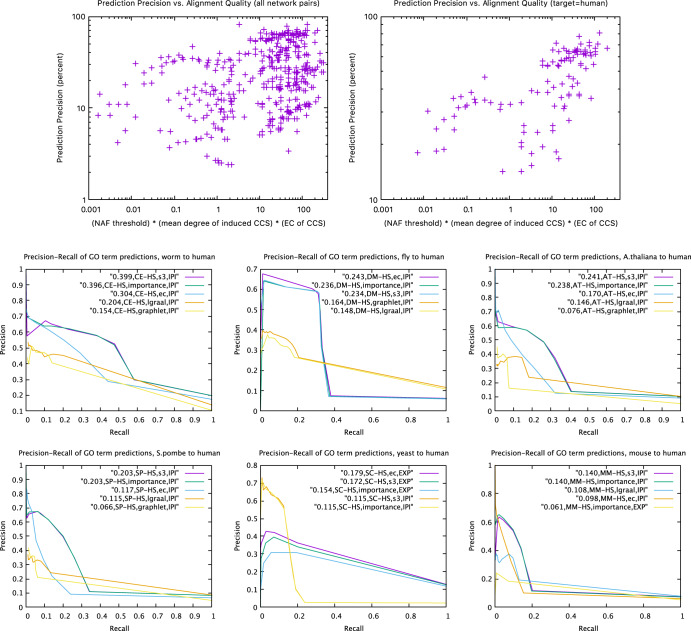
TOP (purple scatter plots): Mean precision of GO term predictions *vs*. alignment quality between all species pairs (left, Pearson *ρ* = 0.19, *p* = 0.002, *n* = 511) and species pairs when human was the target (right, Pearson *ρ* = 0.61, *p* = 2 × 10^−11^, *n* = 107). In both cases, alignment quality is measured as the product of NAF, EC, and mean degree of aligned nodes induced on the CCS with the given NAF. Predictions were made using only BioGRID networks and GO terms available as of April 2010, and validated against GO terms available a decade later (April 2020). Bottom: Precision-Recall of 2010-based NAF predictions of GO annotations for human proteins: Predictions are made using only data available as of April 2010, validated against the GO release of April 2020. We omit any predictions in which the aligned proteins had any known orthology or detectable sequence similarity. We plot precision vs. recall of predictions from global network alignments between the network pairs where human is the target and which satisfy the degree-3 criterion, which are (left to right, top to bottom) *C. elegans, D. melanogaster, A. thaliana, S. pombe, S. cerevisiae*, and *M. musculus*; the six figures are ordered by species from best-to-worst by the mean AUPR of each figure. Within each figure, the legends are ordered best-to-wost by AUPR, and labeled by: AUPR, species pair, measure of topological similarity, and predicting evidence code (i.e., the evidence code of the non-human protein used to source the prediction for the aligned human protein). Precision is the number of correct predictions as a fraction of all predictions arising at the threshold NAF, and the denominator of Recall is the cardinality of the set resulting from the intersection of the following two sets: Predictable in principle pairs, and the annotations actually present in the April 2020 GO release (called the validating set—see “Methods”). See Table 4 for *F*-scores.

The outline of our paper is as follows: we describe Gene Ontology annotations including which evidence codes we deem as “involving sequence” (cf. Table 1 (bottom)), and introduce network alignment (cf. Fig. 1) and the various measure of topological similarity that we employ. We then define NAF—Network Alignment Frequency—which is a measure of confidence for the alignment of each protein pair output by our alignment algorithm SANA^38^. Figure 2 then demonstrates that NAF correlates with Resnik semantic similarity, while the large middle table of Fig. 2 shows that the correlation is especially strong when restricted to proteins that are well-annotated. One of our most important results is demonstrated in Fig. 3: the distribution of Resnik similarity scores of network-aligned protein pairs is independent of whether the pair possess sequence similarity. In other words, NAF uncovers semantic similarity that is invisible to sequence-based methods. Supplementary Table 1 lists the most dense regions of our network alignments, sorted by mean degree, while Tables 2 and 3 demonstrate that prediction precision correlates strongly with NAF in the regions with highest mean degree. Figure 4 (bottom) presents AUPR curves for all 2010-based predictions of human GO annotations validated in 2020; Table 4 and Supplementary Table 2 provide the associated F* measures. Finally, Tables 5 and 6 detail novel predictions of human GO terms based on information available in 2018 and manually validated by literature search.

**Table 1:** TOP: BioGRID (version 3.4.164, downloaded Sept. 2018), sorted by number of edges.

Species	ShortName	Common name	Nodes	Edges	Mean degree	Max degree
*H. sapiens*	HS	Human	17,200	282,181	32.8	2385
*S. cerevisiae*	SC	Baker’s yeast	5984	104,962	35.1	3603
*D. melanogaster*	DM	Fruit fly	8728	46,364	10.6	266
*A. thaliana*	AT	Water cress	9364	34,725	7.42	1341
*M. musculus*	MM	Mouse	6777	18,108	5.34	1671
*S. pombe*	SP	Fission yeast	2811	8931	6.36	298
*C. elegans*	CE	Round worm	3194	5572	3.49	181
*R. norvegicus*	RN	Rat	2391	3554	2.97	808

Code	Description of sequence-based evidence (i.e., disallowed in our predictions)
IBA	curated transfer amongst related sequences Based on common Ancestry (derived by sequence comparison)
IEA	Electronic Annotation (strong sequence-based evidence not directly traceable to experimental evidence)
ISM	Inferred from sequence model
ISA	Inferred from sequence alignment
ISO	Inferred from Sequence Orthology
IGC	Inferred from Genomic Context
RCA	Inferred from Reviewed Computational Analysis
ISS	Inferred from Sequence or Structural Similarity

The graphs are undirected; duplicate edges, self-loops and all interactions with proteins outside the specified species were removed.

BOTTOM: Sequence-based GO evidence codes disallowed in “NOSEQ” cases: Note that we are rather more Draconian in our interpretation of “sequence-based” than is the norm: we disallow any code in which sequence could have had any influence, including manually curated sequence comparison. This supports our hypothesis that NAF discovers semantic similarity “in the absence of sequence similarity”.

**Table 2:** Prediction precision by evidence code and NAF threshold with *H. sapiens* as the target.

Pair	Measure	NAF	#pred	PIP	#val	Precision
*Evidence code EXP*						
SC-HS	EC	2	383	1903	159	41.5%
SC-HS	EC	4	73	1903	30	41.1%
SC-HS	*S*^3^	2	399	1903	150	37.6%
SC-HS	*S*^3^	4	68	1903	22	32.4%
SC-HS	Importance	2	412	1903	125	30.3%
SP-HS	EC	2	49	1906	22	44.9%
SP-HS	EC	4	7	1906	3	42.9%
*Evidence code IPI*						
SC-HS	ec+s3+Imp	4	10,917	19278	2305	21.1%
SC-HS	ec+s3+Imp	7	19,075	19,278	6137	32.2%
SC-HS	ec+s3+Imp	11	12,341	19,278	5436	44.0%
SC-HS	ec+s3+Imp	17	8568	19,278	4655	54.3%
SC-HS	ec+s3+Imp	29	2961	19,278	1838	62.1%
AT-HS	ec+s3+Imp	2	10,600	7740	3471	32.7%
AT-HS	ec+s3+Imp	4	6757	7740	3304	48.9%
AT-HS	ec+s3+Imp	7	3416	7740	1945	56.9%
AT-HS	ec+s3+Imp	11	1564	7740	937	59.9%
AT-HS	ec+s3+Imp	17	610	7740	393	64.4%
AT-HS	ec+s3+Imp	29	109	7740	79	72.5%
CE-HS	ec+s3+Imp	2	8652	3703	4200	48.5%
CE-HS	ec+s3+Imp	4	4482	3703	2603	58.1%
CE-HS	ec+s3+Imp	7	1998	3703	1253	62.7%
CE-HS	ec+s3+Imp	11	637	3703	410	64.4%
CE-HS	ec+s3+Imp	17	133	3703	88	66.2%
CE-HS	ec+s3+Imp	29	15	3703	10	66.7%
DM-HS	ec+s3+Imp	2	18,858	8439	5701	30.2%
DM-HS	ec+s3+Imp	4	10,119	8439	5473	54.1%
DM-HS	ec+s3+Imp	7	6310	8439	3566	56.5%
DM-HS	ec+s3+Imp	11	7546	8439	4358	57.8%
DM-HS	ec+s3+Imp	17	3360	8439	2025	60.3%
DM-HS	ec+s3+Imp	29	59	8439	43	72.9%
SP-HS	ec+s3+Imp	2	8244	6974	2755	33.4%
SP-HS	ec+s3+Imp	4	3026	6974	1669	55.2%
SP-HS	ec+s3+Imp	7	1677	6974	1054	62.9%
SP-HS	ec+s3+Imp	11	786	6974	529	67.3%
SP-HS	ec+s3+Imp	17	347	6974	235	67.7%
SP-HS	ec+s3+Imp	29	71	6974	53	74.6%
MM-HS	ec+s3+Imp	2	8469	13394	3191	37.7%
MM-HS	ec+s3+Imp	4	2629	13394	1489	56.6%
MM-HS	ec+s3+Imp	7	849	13394	544	64.1%
MM-HS	ec+s3+Imp	11	239	13394	148	61.9%
MM-HS	ec+s3+Imp	17	31	13394	13	41.9%
MM-HS	ec+s3+Imp	29	8	13394	5	62.5%

This table summarizes prediction precision as a function of NAF for species aligned with human satisfying the degree-3 criterion. The species pairs are presented in order of mean CCS degree, highest to lowest (cf. Supplementary Table 1). We show predictions based on source evidence codes EXP (top section) and IPI (bottom section) available in 2010 and validated (with any evidence) in 2020. PIP means predictable in principle, and refers to the absolute maximum number of predictions that would be possible in principle given the information available as of April 2010 (see text). To save space in the IPI case, we have conglomerated the measures EC, *S*^3^, and Importance, since all three had similar validation rates at fixed NAF (within 10% of each other in all cases). Also to save space, not all values of NAF are listed here, but the Pearson correlation between NAF and precision across all NAF values are presented below, in Table 3.

**Table 3:** Correlation between NAF and prediction precision for each species pair, across rows similar to those in Table 2.

Pair	Rows	*ρ*	*p*-value	*σ*
SC-DM	18	0.8436	3.416 × 10^−5^	6.2844
SC-HS	17	0.8741	8.544 × 10^−6^	6.9706
MM-DM	14	0.709	0.0356	3.4823
SP-DM	18	0.5425	0.1937	2.5832
AT-HS	17	0.8338	0.0001243	5.8490
AT-DM	17	0.9301	1.284 × 10^−8^	9.8053
CE-HS	16	0.6352	0.07426	3.0771
MM-CE	16	0.9252	1.032 × 10^−7^	9.1228
MM-SP	16	0.7642	0.003988	4.4333
SP-AT	18	0.8393	4.496 × 10^−5^	6.1739
DM-HS	16	0.7672	0.003627	4.4753
AT-CE	17	0.8804	5.087 × 10^−6^	7.1898
SP-CE	18	0.8864	1.15 × 10^−6^	7.6598
CE-DM	15	−0.2407	0.4805	0.8943
SP-HS	17	0.7413	0.005154	4.2774
MM-HS	15	0.2273	0.5011	0.8418
RN-CE	16	0.7	0.02165	3.6677
MM-AT	14	0.9327	4.893 × 10^−7^	8.9604
RN-SP	6	0.8793	0.08192	3.6916
RN-DM	6	0.8885	0.06864	3.8726
RN-AT	6	0.9643	0.003344	7.2840
RN-MM	6	0.9068	0.04537	4.3019
TOTAL (non-normalized)	213	0.314	0.00023	4.74
TOTAL (normalized)	213	0.749	7.27 × 10^−38^	15.5

Each row represents one species pair with the NAF-precision correlation across all measures. The second-last row is the correlation between NAF and prediction precision across all species and all measures. However, as seen in Table 2, the scaling between NAF and precision can differ substantially across species, which muddles the correlation. We correct for this in the final row, where we have normalized the NAF and precision to their maximum values on a per-species basis.

**Table 4:** 2010-based predictions ranked by F*.

Rank	F*	NAF	Pair	*M*	EvCode	$| {P}_{12}\cap {\Gamma }_{2}^{\prime}|$	pred	Valid	Precision
1	0.500	2%	CE-HS	*S*^3^	IPI	3443	3131	1643	52.5%
2	0.496	2%	CE-HS	Import.	IPI	3443	3146	1634	51.9%
3	0.424	16%	SP-AT	Import.	IPI	455	569	217	38.1%
4	0.417	16%	SP-AT	*S*^3^	IPI	455	577	215	37.3%
5	0.416	5%	DM-HS	*S*^3^	IPI	4080	3237	1521	47.0%
6	0.404	5%	DM-HS	EC	IPI	5978	3204	1855	57.9%
7	0.375	8%	SP-AT	EC	IPI	472	968	270	27.9%
8	0.358	2%	CE-HS	EC	IPI	3443	2127	998	46.9%
9	0.346	2%	AT-HS	*S*^3^	IPI	5038	4360	1627	37.3%
10	0.341	2%	AT-HS	Import.	IPI	5038	4365	1605	36.8%
11	0.340	8%	MM-AT	*S*^3^	IPI	725	768	254	33.1%
12	0.309	5%	DM-HS	Import.	IPI	9018	3245	1895	58.4%
13	0.298	2%	SP-HS	*S*^3^	IPI	5380	4022	1401	34.8%
14	0.298	2%	SP-HS	Import.	IPI	5380	3985	1394	35.0%
15	0.291	5%	MM-AT	EC	IPI	755	1043	262	25.1%
16	0.277	1%	CE-HS	lgraal	IPI	3140	1511	644	42.6%
17	0.260	8%	SP-AT	graphlet	IPI	455	808	164	20.3%
18	0.259	8%	SP-AT	lgraal	IPI	472	827	168	20.3%
19	0.257	1%	SC-HS	EC	EXP	2111	1158	420	36.3%
20	0.249	2%	AT-HS	EC	IPI	5038	3629	1080	29.8%

NAF: threshold that achieved F*; pair: species pair (cf. Table 1 (top)); M: topological measure; EvCode: evidence code supporting the annotation of the source (non-human) protein that produced the predicted human protein annotation; $| {P}_{12}\cap {\Gamma }_{2}^{\prime}|$: intersection of the number of predictable in principle annotations (*P*_12_, see “Methods”) with ${\Gamma }_{2}^{\prime}$—all annotations available in the validation set. pred: number of predicted annotations made using the specified source evidence code at the specified NAF (note this number can be bigger than the previous column since, clearly, any number of predictions can be made that may not appear in the validating GO release). valid: the number of validated predictions by any non-sequence-based evidence code. Precision: number of predictions that were validated.

**Table 5:** All zero-sequence-similar cilia-related GO term predictions from BioGRID mouse to human with NAF 8% or greater: NAF is the network alignment frequency at which the Mouse protein was aligned to the Human protein.

NAF	Mouse	Human (& aliases)	T	Predicted cilia-related GO: term(s)	Species+validation
16	MKS1	HDAC5 CLUH	P	1905515, 0044458, 0060271, 0060122,	F^63,65^ M,H^64^
16	MKS1		C	0005929, 0036064, 0035869	
15	NPHP1		C	0031514, 0005929, 0035869, 0097546, 0032391	
12	AHI1		P	1905515, 0060271	
12	AHI1		C	0005929, 0036064, 0097730	
13	SHANK3	CAND1	C	0060170	H^66^
12	SHANK3	RPL6 IRS4	C	0060170	H?^67^
12	IQCB1	CUL7 RAD51C	P	0060271	H^68^
12	IQCB1		C	0032391	
11	NPHP4	POLR3D CFTR	C	0005929, 0036064, 0035869, 0097546, 0032391, 0097730	H^69–71^
10	APP	CDH1	C	0035253	H^72^?^73^
10	SHANK3	EEF1A2 HNRNPU	C	0060170	M?^74^
10	SHANK3	RPL18 ITGA5	C	0060170	R?^76^
9	IQCB1	RNF2	P	0060271	H^77,78^
9	IQCB1		C	0032391	
9	FAM92A	VCAM1	P	0060271	M^79^
9	FAM92A	VCAM1	C	0036064, 0097546	
8	IQCB1	VCAM1	P	0060271	
8	IQCB1	VCAM1	C	0032391	
9	NPHP4	MUC4 XPO1	C	0005929, 0036064, 0035869, 0097546, 0032391, 0097730	M^80^
8	DYNC1H1	RPS9	P	0003341	F^87^
8	HTT	CUL5 NUP50	P	1905505, 1902857, 0045724	H^81^
8	AHI1	C1ORF87	P	1905515, 0060271	H^88^
8	AHI1		C	0005929, 0036064, 0097730	
8	NPHP1	CCDC8	C	0031514, 0005929, 0035869, 0097546, 0032391	M,H^82^
8	FAM92A	HIAT1 OBSL1	P	0060271	M,H^85^
8	FAM92A		C	0036064, 0097546	
8	NPHP3	LMOD1 SOD1	P	1905515, 0060271	M^86^
8	NPHP3		C	0005929, 0097543, 0097546	
8	EPS8	RPLP0P6	C	0032426, 0032420, 0032421	Not validated
8	EPS8	CNBP MYCL	C	0032426, 0032420, 0032421	H^89^

In all cases, the mouse protein was annotated with the specified GO terms but the human protein was not (even indirectly). T is “type” (P = Biological Process, C = Cellular Component) of the predicted GO terms; predicted GO terms are listed with the leading “GO:” and leading zeros removed; Species+Validation lists the species (H = human, M = mouse, R = rat, F = fish) for which cilia-related activity for that protein has been validated, along with the reference for the corroboration—however, in all cases the authors of the citations strongly implied that their results were applicable to humans, though a question-mark indicates the evidence was weak or only implicit.

**Table 6:** GO terms present in Mouse Fancd2 but not Human Trim25, along with the global frequency of the GO term, the evidence code, the GO Category (Biological Process, Cellular Component), and description.

GO term	freq	Evidence	Cat	Description
GO:0005634	14,731	IDA	C	Nucleus
GO:0034599	285	IGI	P	Cellular response to oxidative stress
GO:0000793	100	IDA	C	Condensed chromosome
GO:0048854	55	IGI	P	Brain morphogenesis
GO:0097150	47	IGI	P	Neuronal stem cell population maintenance
GO:0006974	673	IMP	P	Cellular response to DNA damage stimulus
GO:0050727	84	IMP	P	Regulation of inflammatory response
GO:0007129	72	IMP	P	Synapsis
GO:0007276	44	IMP	P	Gamete generation
GO:0051090	29	IMP	P	Regulation of DNA-binding transcription factor activity
GO:0045589	20	IMP	P	Regulation of regulatory T cell differentiation
GO:2000348	4	IMP	P	Regulation of CD40 signaling pathway

Top section: Non-IMP-based GO term predictions, sorted from most general (high frequency in the 2nd column) to most specific (low frequency). Bottom section: GO terms predicted by the IMP evidence code, for which we did not attempt literature validation due to time constraints.

## Results

### Global network alignment

We focus on the Pairwise Global Network Alignment (PGNA) problem: pairwise, because we align exactly two networks *G*_1_ and *G*_2_ that have *n*_1_ and *n*_2_ nodes, and we assume without loss of generality that *n*_1_ ≤ *n*_2_; global, because we aim to find a mapping from every node in *G*_1_ to some node in *G*_2_; and network (as opposed to sequence) alignment because we aim to use only the network connectivity information (aka global network topology) to guide creation of the network alignment (see “Methods” for a formal definition). Figure 1 depicts a schematic diagram of a small PGNA.

### Network alignment quality measures

To demonstrate a relationship between network topology and semantic similarity, we start by elaborating on how each is measured.

#### Semantic similarity between pairs of individually aligned proteins

Given a pair of proteins *p* ∈ *G*_1_, *q* ∈ *G*_2_, we measure their semantic similarity using the “maximum” variant of Resnik Semantic Similarity^41,42^ applied to Gene Ontology (GO) terms^43^ as implemented by the Python package FastSemSim^44^. Every GO term that annotates a gene or protein has an associated evidence code describing the evidence that backs the annotation. Most evidence codes are either based directly on experiment, or inferred through some mechanism. Some mechanisms for inferring GO annotations include sequence analysis. Since one of our main goals is to demonstrate that NAF can highlight Resnik similarity in the absence of sequence similarity, we distinguish between Resnik values that allow all types of evidence (“allGO”) vs. those that disallow any evidence based on sequence ("NOSEQ”). Table 1 (bottom) lists the evidence codes we disallow in the latter case.

#### Topological similarity of a global alignment between two networks

The topological similarity of an alignment between two networks can be scored in many ways, including quantifying edge overlap^17,45,46^, node “importance”^47^, graphlet similarity^16,48,49^, graph edit distance^50,51^, and graph spectra^52^. While some work has been conducted to compare how alignment strategies and objective functions each independently affect the biological relevance of an alignment^46,53^, our companion paper^40^ performs the first comprehensive, level-playing field study to compare a large number of topological measures for their ability to recover biological information. Figure 1 provides a schematic example of two purely edge-based measures: EC^17^ (variously called Edge Correctness, Coverage, Correspondence, or Conservation by various authors), and *S*^3^ (the *Symmetric Substructure Score*^45^).

### Statistical sampling of stochastically-generated network alignments using simulated annealing

Anybody who shakes a box of loose items in an attempt to make the contents “settle” already intuitively understands annealing: vigorous shaking re-initializes the system to a new random state, while more refined shaking hones the solution towards a “settled” state which is typically different each time. Crucially, all settled states found by the same “shaking schedule” tend to end with roughly equal energy, even though the final positions of the package contents are different each time. In its essence, our NAF detects pairs of proteins whose alignment is repeatable across multiple, independent, stochastically generated, near-optimal alignments.

#### Network alignment frequency (NAF)

We say that a pair of proteins that appears frequently in well-scoring topological alignments have a high propensity to align together. For each of the 28 pairs of BioGRID networks from Table 1 (top), we independently generate 100 alignments, each driven to optimize the same objective function for a 1 h run of SANA. (All runs used a 24-core Intel X5680 CPU running at 3.33 GHz with 96 GB of RAM.) We chose 1 hour because that was the shortest run that produced objective function values within a few percent of the asymptotic value of much longer runs^40^. Once the 100 runs are finished, we count the frequency (minimum zero, maximum 100) that each pair of aligned nodes appears across the 100 network alignments. The result is NAF: a node-by-node output measure *ϕ*_*pq*_, which is the frequency, or propensity, of alignment between proteins *p* ∈ *G*_1_, *q* ∈ *G*_2_. The higher the frequency, the higher the propensity for alignment between *p* and *q*. Note that while many measures exist^16,17,21,46,48,49,52,54^ for computing topological similarity between two nodes *p* ∈ *G*_1_, *q* ∈ *G*_2_, they are all pre-computed and provided as input to the alignment process, remaining constant throughout the process. Ours is the first topology-only network alignment method to produce a pair-by-pair score as output.

The network alignment frequencies generated above by multiple runs of SANA are a generalization of core alignments, that were introduced by GRAAL^17^ and developed further by H-GRAAL^19^. GRAAL used randomness only to break ties while building an alignment greedily using graphlet measures, while H-GRAAL used the Hungarian Algorithm to exhaustively enumerate all optimal solutions to a given graphlet-based local measure. In both cases, it was observed that there were a subset of aligned protein pairs (the “core”) that appeared in all optimal alignments, and that the mean semantic similarity measured across this core of always-aligned protein pairs was higher than protein pairs whose alignment partners changed between alignments. NAF simply generalizes this idea to stochastically generated network alignments that have been optimized to maximize some measure of global topological similarity.

We note that even though SANA produces only 1-to-1 network alignments on each individual run, the merged output of *N* such alignments effectively produces many-to-many network alignments, with the added value of an output score for each possible pair of nodes. This merging of multiple network alignments also alleviates a potential problem called “low alignment coverage.” In particular, it has been noted^55^ that 1-to-1 network alignment algorithms do not provide alignment suggestions for all possible nodes in both networks. Their solution was to combine the outputs of several algorithms in order to improve this coverage. However, our network alignment frequency measure makes this unnecessary, since every possible pair of nodes can be assigned an approximate propensity value; pairs that never appear are simply assigned an approximate propensity of zero.

### Correlation between semantic similarity and NAF

For each value *ϕ* of NAF, the mean Resnik similarity was computed across all aligned protein pairs with at least frequency *ϕ*. We then plotted the Resnik values of various subsets of pairs allowing various subsets of GO evidence codes. We will depict our results split across three “axes”: (a) which topological objective was being optimized (our two examples here being EC^17^ and graphlet-GDV^16^); (b) whether or not the aligned node pair possess sequence similarity; and (c) whether we allowed the use of sequence-based GO evidence codes when computing the Resnik score. Before studying the details, we first draw attention to our primary conclusion: when the aligned pair of proteins possess sequence similarity, then sequence-based evidence codes provide a “boost” to the Resnik score; conversely, this boost is impossible for aligned pairs of proteins that do *not* possess sequence similarity, resulting in a potential bias towards a low Resnik score for such pairs. We stress that the separation of aligned protein pairs into those that do, or do not, possess sequence similarity is done after the fact: sequence plays absolutely no role in creating our alignments or computing NAF. The sequence of events is (1) create 100 alignments by optimizing a topology-only objective function; (2) compute NAF for each pair of aligned proteins observed in the 100 alignments; (3) compute two Resnik scores for each pair of aligned proteins: one that allows the use of sequence-based evidence codes, and one that does not; (4) finally, once all scores are fixed (both NAF and Resnik), separate the aligned protein pairs into two groups: those that possess sequence similarity, and those that do not.

Figure 2 plots Resnik similarity *vs*. NAF in 12 “postage-stamp” sub-figures, arranged with the top row of postage stamps depicting alignments between mouse (MM) and human (HS), and the bottom row between yeast (SC) and human. In each row, the left three postage stamps (which we call a “column-triplet”) depict alignments that were driven to optimize EC, while those in the right column-triplet were driven to optimize Graphlet Degree Vector Similarity^16^. Each “postage stamp” displays the mean (blue line) and standard deviation (blue shaded area) of Resnik semantic similarity (measured on the left axis with scores from 0 to 12) between pairs of individually aligned proteins as a function of NAF. The red line (right axis, logarithmic from 1 to 10^5^) depicts the number of pairs that aligned with that NAF or higher. Within each column-triplet, the three columns depict:

(left) only those aligned protein pairs that possess sequence similarity, and for which we allowed sequence-based evidence codes in the Resnik score (column labeled at the bottom with “seqSim/allGO”);

(mid) all aligned protein pairs, again allowing sequence-based evidence (column “allPairs/allGO”);

(right) all aligned protein pairs, but disallowing sequence-based evidence codes (column “allPairs/GO-NOSEQ”).

Note that the latter two columns of each column-triplet in Fig. 2 depict the same set of aligned node pairs, the only difference being that the former allows sequence-based evidence codes while the latter does not. Conversely, the first column of each triplet lists only those pairs that actually possess sequence similarity (see “Methods”).

In each column-triplet of Fig. 2, comparing the three postage stamps reveals, respectively, that (1) allowing sequence-based evidence significantly enhances the measured Resnik similarity, but obviously only for that minority of pairs that actually *possess* sequence similarity; (2) the sequence-similar pairs and their sequence-based evidence enhance the mean Resnik similarity across all aligned pairs, over the Resnik value obtained when (3) no sequence-based evidence is allowed. (In the cases that the semantic similarity trend reverses and starts to decrease with alignment frequency, it is usually when the number of aligned pairs is below 30, which can be attributed to statistical noise.) Comparing the two objective functions, we see that EC achieves maximum NAF frequencies of about 15–20 with mean Resnik scores of about 4–8 (depending on whether we allow sequence-based evidence). In contrast, the graphlet-GDV objective provides hundreds of aligned pairs with very high NAF (up to 100), though their Resnik scores are significantly lower on average. We will see below that even with these apparent low scores, graphlet-based objectives still retain significant predictive power. Supplementary Fig. 1 shows that NAF correlates well with Resnik similarity even when we separate GO terms based on biological process (BP), cellular component (CC), and molecular function (MF), across all aligned pairs and allowing all GO terms.

We move now to the tables below the postage stamps of Fig. 2. The top table lists the Pearson correlations and statistical significance of the plots. For the species pairs mouse-human (“MM-HS”, top two rows) and yeast-human (“SC-HS”, bottom two rows), we list the number of aligned protein pairs (“#aligs”) with NAF score 2% or more, and compute the Pearson correlation between NAF and Resnik using either GO terms of all evidence codes (middle section) or excluding any GO terms with sequence-based evidence, even if they also have non-sequence evidence (right section). In each section we list the Pearson correlation *ρ*, the *p*-value computed using Fisher’s *r*-to-*z* transformation, as well as the number of standard deviations (*σ*’s) from random represented by that *p*-value.

The correlations (*ρ* values) listed in the top table of Fig. 2 are on the low side. The primary reason for this is due to lack of GO information: the majority of proteins have few GO annotations, or only very vague ones. The mathematical formulation of the Resnik score^41,42^ requires that both proteins be well-annotated to achieve a high score. This fact is clearly demonstrated in Supplementary Fig. 4, where we see that the Resnik score between a pair of proteins clearly increases as the number of annotations on the less-well-annotated protein increases. For example, if only a small fraction *ε* of proteins are well-annotated by some criterion, then only a fraction ≈ *ε*^2^ of protein pairs will be well-annotated by the same criterion. Luckily, our 100 alignments provide us with about half a million pairs of aligned proteins for any given pair of species—more than enough to allow us to filter for well-annotated pairs. (If both proteins are well-annotated but with very different annotations, then they will have a meaningful low score, as opposed to a low score due to lack of information.)

To account for this, we now filter protein pairs for annotation quality. First, note that a GO term’s specificity is inversely proportional to how many proteins it annotates: GO terms that annotate only a few proteins tend to provide more specific information than vague GO terms that annotate thousands of proteins. Furthermore, proteins annotated with highly specific GO terms tend to be better understood than those that are not. In the large middle table of Fig. 2, each row displays the correlation between NAF and Resnik after filtering for well-annotated protein pairs. In particular, for a given row labeled with (*S*, *M*) in the last two columns, a protein pair is included only if each protein is independently annotated by at least *S* distinct GO terms each of which annotates at most *M* proteins per species. The table exhaustively lists every statistically significant (*p* < 0.01) correlation observed for *S* ≥ 8 and *M* ≤ 4 optimizing the EC objective, sorted by *ρ*(*S*, *M*). For example, the top row depicts alignments between the species pair CE-DM (worm *C. elegans* and fruit fly *D. melanogaster*); although not depicted, the 100 CE-DM alignments contained exactly 302,169 distinct protein pairs with non-zero NAF; among these, there were only *N* = 23 in which both proteins were annotated by at least *S* = 16 distinct GO terms each of which annotated at most *M* = 1 proteins in its respective species. In other words, these 23 protein pairs are very well understood—they each possess least 16 GO terms that uniquely annotate *that* protein and no other in its species. In this case, we see that correlation between NAF and Resnik is *ρ*(*S*, *M*) = 0.661—much higher than the correlations seen among the unrestricted protein pairs in the table immediately above it.

The large middle table of Fig. 2 lists only a small subset of (*S*, *M*) values we tested, which included all pairs where *S* and *M* independently ranged from 1 to 1024 in powers of 2 (10 values each), for both the *EC* and graphlet measures—200 rows per species—across all ${5\choose2}=10$ pairs of the 5 best-annotated BioGRID species: *C. elegans, D. melanogaster, M. musculus, S. cerevesia,* and *H. sapiens* (CE, DM, MM, SC, and HS, respectively). Merging all of these cases gives a table with 2,000 rows, each one with a NAF-Resnik Pearson correlation *ρ*(*S*, *M*) and *p*-value. Of particular interest is what happens when we compute the Pearson correlation between *ρ*(*S*, *M*) and either *S* or *M*. More formally: Given a pair of species *s*_1_, *s*_2_ and values of *S* and *M* each ranging from 1 to 1024 in powers of 2, let *ρ*(*S*, *M*) refer to the Pearson correlation between NAF and Resnik restricted to protein pairs satisfying the *S*, *M* requirements. Out of the 2000 rows, there are 1599 in which the correlation *ρ*(*S*, *M*) is statistically significant (*p* < 5 × 10^−6^, chosen to ensure a statistical significance of at least *p* < 0.01 after Bonferroni correction across 2000 rows); we find that *ρ*(*S*, *M*) is itself correlated with each of *S* and *M*, independently. Since this is a correlation of correlations, we refer to it as a Pearson^2^. Observing the bottom table of Fig. 2, we see that there is a strong and highly significant correlation with *M* (negative because specificity increases as *M* decreases), and separately a strong and highly significant positive correlation with *S* (the number of such GO terms possessed by both proteins). The correlation becomes even stronger if we use *S*/*M* as the independent variable. In English, the bottom table of Fig. 2 demonstrates that the more we know about two proteins that have been aligned, the better the correlation between their alignment frequency (NAF) and their mutual Resnik score. This observation suggests that high NAF scores tend to uncover protein pairs with genuine high similarity, even if that similarity is not (yet) well-documented with GO terms; in turn, this suggests that NAF can be used as a measure of confidence that two proteins possess GO-based semantic similarity.

### The NAF-function postulate

In each column-triplet of Fig. 2, the second and third columns (“allPairs”) show significantly lower Resnik scores than the first, which plots only pairs that possess sequence similarity according to BLAST (bitscore threshold 13, E-values allowed from 0 to 1000). Since NAF aligns protein pairs based only on similar network topology, and the tables of Fig. 2 strongly support the hypothesis that NAF correlates with Resnik semantic similarity, we propose the following:

NAF-function Postulate: *protein pairs aligned at or above a given Network Alignment Frequency (NAF) are drawn from a single distribution of functional similarities, regardless of whether or not they possess significant sequence similarity*.

We provide evidence for the NAF-function Postulate below, but if true, it suggests that, compared to the first column of each column-triplet in Fig. 2, the lower scores of the second and third columns is spurious, because allowing GO terms derived from sequence-based evidence will only benefit that minority of protein pairs that actually possess sequence similarity; those pairs that do not possess sequence similarity cannot benefit from sequence-based evidence that does not exist. Of course, we are not claiming that sequence-based evidence is untrustworthy; it is simply inapplicable to protein pairs that do not possess sequence similarity. If one assumes that the Resnik scores in the left column (“seqSim/allGO”) are indicative of true similarity for protein pairs at a particular NAF, then the NAF-Function Postulate asserts that the Resnik scores in the second and third columns are artificially low. In essence, the NAF-function postulate states: *sequence-based evidence doesn’t help when it doesn’t exist—but absence of evidence is not evidence of absence*. This, combined with the already-known fact that functional similarity can exist despite little or no detectable sequence similarity^1–3^, makes the NAF-function postulate a plausible extension of existing knowledge.

We now provide evidence for the NAF-function Postulate. First, to apply a level-playing-field comparison of Resnik similarity between pairs of nodes that may or may not share sequence similarity, we disallow the use of sequence-based evidence in computing the Resnik score (cf. Table 1 (bottom)). Surprisingly, even after removing sequence-based evidence, sequence-similar proteins retain a significant Resnik advantage at fixed NAF. Careful investigation reveals that proteins with sequence similarity tend to be better-annotated even with non-sequence evidence than those without (Supplementary Fig. 3). While the reason behind this bias in annotation levels is beyond the scope of this paper (popularity?^13^), the effect is easily removed by accounting for level of GO annotation. Figure 3 plots the mean Resnik score as a function of annotation level (i.e., number of GO terms, disallowing sequence-based evidence), across all aligned protein pairs with NAF 2% or more. After separating those aligned protein pairs with, and without, sequence similarity, we observe that the two curves are statistically indistinguishable, suggesting that sequence similarity plays little or no role in the NAF-Function Postulate. In other words, while high sequence similarity is often sufficient to infer functional or semantic similarity, it is by no means necessary: removing sequence-based evidence and comparing the Resnik similarity between protein pairs at equal annotation level, the impact of sequence similarity on semantic similarity is negligible. More to the point, this suggests that when two proteins without sequence similarity are aligned with NAF at or above some threshold *ϕ*, their semantic similarity tends to be about the same as equal-NAF pairs with sequence similarity. While Fig. 3 only demonstrates this for *ϕ* = 2, the previous sentence equates to the NAF-Function Postulate.

Finally, we note the obvious fact that protein pairs with high sequence similarity are rare among the space of all protein pairs, which is why—when it occurs—sequence similarity correlates well with semantic similarity. Similarly, protein pairs with high topology-based network similarity (as quantified by NAF) are also rare in the space of all protein pairs, and that network similarity correlates equally well with semantic similarity. Figure 3 establishes that topological network similarity also correlates with functional and semantic similarity, *independent* of whether the topologically-aligned protein pairs share sequence similarity.

### NAF predicts common GO terms even in the absence of sequence similarity

The bottom two tables in Fig. 2 show than when both proteins are well-annotated, we observe a strong positive correlation between NAF and the demonstrable similarity between the pair of proteins aligned. This suggests that NAF can be used as a measure of confidence that two proteins share some common set of GO terms: if two proteins are aligned with high NAF but only one of them is well-annotated, there is a basis for using the GO terms possessed by one as predictions of GO terms possessed by the other, with NAF providing a measure of confidence of the predictions. Here we test this hypothesis in several ways.

#### Predictions from the year 2010, validated today

To demonstrate that NAF’s success is not simply due to the greater amount of network data available today than previously, we have performed the required 100 alignments on the same species as in Table 1 (top), but using BioGRID 3.0.64, released on 23 April 2010. We then used the Gene Ontology release of the same month to predict novel (as of April 2010) GO annotations between species, as follows: Let *p*_*g*,*e*_ represents the fact that protein *p* is annotated with GO term *g*, supported by evidence code *e*. For each pair of proteins (*p*, *q*) aligned by SANA with NAF ≥ *ϕ*, assume we wish to use the GO terms of *p* (the “source”) to predict those of *q* (the “target”). For each GO term *g* from the source protein *p*, and for each evidence code *e* relating *p* to *g*, we increment a counter *q*_*g*,*e*_ by *ϕ*. Note that this allows GO terms and their evidence codes for target *q* to accumulate across different proteins *p* of the source species—essentially, if *q* is aligned with multiple proteins *p* and all of these alignment partners agree than *q* should be annotated with GO term *g*, then the NAF value accumulates across all such *p*’s. For example, if a GO term appears among multiple non-human proteins each aligned with the same human one, all contribute to the score of the human protein being annotated with that GO term, with that evidence code. At the end, we have a cumulative score for *q* being annotated with *g*, across various evidence codes *e*. If the cumulative score is above a pre-specified threshold Φ (used in precision-recall calculations, see “Methods”), it counts as a prediction. We then validate these predictions by checking to see if the predicted GO term shows up as annotating the human protein in a later release of the GO database. We find that the validation rate depends heavily on the evidence code used to justify the annotation of the non-human protein. By far the evidence codes with the greatest predictive power (from 2010) are IPI (Inferred from Physical Interaction), EXP (experimentally determined), and IDA (Inferred from Direct Assay), in that order. (Keep in mind that these are the evidence codes for the source protein—the non-human one.) This resulted in over 3000 novel annotations to almost 4000 human proteins, including 137 human proteins that had zero GO annotations as of April 2010.

We made every effort to exclude annotations that could have been either predicted, or validated using any form of sequence information. In particular, we eliminated from consideration (1) any pair of proteins that had sequence similarity according BLAST (used with its default parameters resulting in bit scores of 13 or higher); and (2) any pair of proteins listed as orthologs—even distant ones—in any of NCBI Homologene, InParanoid 8^56^, or the 2019 release of EggNog^57^. In addition, we excluded any GO annotation that was supported by any sequence-based evidence code, even if it also had non-sequence-based evidence. Finally, this procedure was applied both to the 2010 GO release from which GO term predictions were sourced, as well as 2020 GO release which was used to validate predictions. Though these conditions are likely more stringent than one would want in a production-level prediction pipeline, our goal here is to demonstrate that none of the predictions discussed below could have been made, or even validated, using any form of sequence information. In short, the predictions below should be largely orthogonal to predictions that are based on sequence analysis.

In the process of studying prediction precision, we discovered that some sets of 100 alignments provided few validated predictions even with a high NAF threshold. Investigation revealed that the alignments in question had little topology in common despite the high NAF of the aligned nodes. In particular, given a set of nodes with NAF above a threshold, the Common Connected Subgraph (CCS) is the set of edges in common among the aligned nodes—cf. the purple edges emanating from purple nodes in Fig. 1. We found that prediction precision suffered significantly in two distinct cases. By far the most frequent case was when the mean degree of (purple) nodes of the CCS were low even with high EC or *S*^3^ scores (cf. Fig. 1)—in other words, while most edges were aligned, there simply were not very many of them—possibly meaning the high EC and *S*^3^ were due to chance. Less frequently, we found cases where the mean degree of the CCS was high, but the number of *non*-aligned edges was even higher, making both the EC and *S*^3^ scores low. Figure 4 (top) quantifies this effect by plotting prediction precision vs. “alignment quality” as measured by the product of NAF, and the EC and the mean degree of nodes in the CCS induced with that NAF. Importantly, like NAF, this measure of “alignment qualtity” is computable a priori as part of the alignment output. Since the low-degree case was by far the most frequent cause of low prediction precision, for the purposes of this paper we will arbitrarily apply a lower bound of 3 on the mean degree of the induced CCS to eliminate cases of low prediction precision; we call this the degree-3 threshold, and leave to future work how to more rigorously choose such a bound.

Supplementary Table 1 shows, for each species pair and each measure, the NAF value that achieved the highest mean degree ${\overline{D}}_{max}$ on the resulting induced CCS. Surprisingly, although the edge-based measures EC, *S*^3^, and Importance frequently reach the degree-3 threshold, we observe that the graphlet-based measures rarely result in a mean degree above 1, and never above 3. Table 2 depicts the prediction precision as a function of NAF for all species paired with human (HS), so long as the mean degree of the CCS was above 3; only RN (*Rattus norvegicus*) failed to satisfy the degree-3 threshold (cf. Supplementary Table 1). Observe that in IPI section of Table 2, the prediction precision generally increases with NAF. Table 3 expands on this by showing that the prediction precision almost always has a strong positive correlation with NAF (though in some cases not enough distinct NAF values exist to make the correlation statistically significant, and the one case with a negative correlation is far from statistical significance). These correlations corroborate the hypothesis that higher NAF provides greater confidence that the aligned protein pair share common GO terms.

Armed now with the knowledge of which species pairs have “robust” alignments based on the mean degree-3 threshold of the CCS, Fig. 4 (bottom) presents precision-recall curves using NAF thresholds, across the 6 species aligned with human that satisfied the degree-3 threshold, broken down by predicting evidence code and measure of topological similarity used to drive the alignment. The number of predictions are not depicted, but for example GO terms with IPI evidence in 2010 from yeast and fly produced 2959 and 2050 validated, novel GO annotations of human proteins, respectively; EXP produced 367 and 187, respectively. Other evidence codes for these species had AUPR’s below 0.01, though some other species pairs had non-negligible AUPRs (see Supplementary). Table 4 lists the top 20 sets of predictions across all species pairs satisfying the degree-3 threshold, ranked by F* (best *F*_1_ score), broken down by GO evidence code; Supplementary Table 2 does the same for GO category (Biological Process, Cellular Component, Molecular Function). We see from Table 4 that the most successful evidence code for making predictions is IPI (Inferred through Physical Interaction), while Supplementary Table 2 shows that GO terms in the category Molecular Function are by far the most successfully predicted. These conclusions may change as the date of prediction moves forward.

We note that, even though these predictions are made with 10-year-old networks, our best AUPRs are competitive with the best sequence- and structure-based predictors in the 2017 CAFA3 competition as well as recent algorithms comparing themselves to CAFA3^58–62^. (It is impossible to compare directly against CAFA because no PPI network data is available for the species used in CAFA.) We emphasize again, however, that our predictions were neither made nor validated using sequence information, and so we believe our predictions are orthogonal to those that are possible from CAFA, and thus purely additive to existing prediction methods. Finally, it is interesting to note the high quality of these predictions even though Resnik-NAF correlations are much weaker in 2010 data than in Fig. 2 (Supplementary Fig. 2).

#### Predictions using 2018 data, validated today by literature search

The painstaking effort required to create the Gene Ontology database by human curation of the literature necessarily means that the GO database lags behind knowledge available in the most recent, live literature. Thus, we repeated the same effort as we did for 2010, but using BioGRID 3.4.164 (Sept. 2018, the same release as was used in Figs. 2 and 3), using the GO database of the same month. Our goal is to produce bona fide predictions of GO annotations to human proteins. We expect that the relevance of inter-species GO term predictions will be highest when (a) the two species are as closely related as possible; and (b) both PPI networks are as complete as possible. Thus, we choose to align the human PPI network with that of mouse, since mouse and human are both mammals, and mouse has the most complete mammalian PPI network after human.

All below predictions of the annotation of human protein *p* with GO term *g* are bona fide predictions, in the sense that the annotation of *p* with *g* was not present in the Sept. 2018 GO release, either directly, nor by inference on the GO hierarchy. For reference, out of the ~150,000 GO annotations to human proteins, only 340 (0.23%) contained the word “cilia”; the numbers for mouse were comparable, at 285 out of 110,000 (0.26%).

#### Literature validation of our top cilia-related GO term predictions

To keep our job of manual literature curation tractable, we narrowed the scope to cilia-related predictions from mouse to human with a NAF of 8% or greater, with cilia chosen on the advice of a senior curator of the Gene Ontology Consortium (Karen Christie, personal communication). We use cilia-related GO annotations of mouse proteins to predict the same GO annotations to human proteins lacking such annotations. We avoid all cases that could be related via sequence or orthology—in other words, we omit predictions where the aligned mouse and human proteins had any known orthology or detectable sequence similarity, even if the mouse protein had an annotation that the human one did not. Table 5 shows that these predictions achieve a high rate of literature validation. We stopped at NAF = 8 since lower values of NAF had dozens to hundreds of predictions, which is too many to validate manually.

Below we provide a brief summary of each citation used in Table 5 that was used as evidence of cilia-related activity. There are 19 distinct human proteins with predicted cilia-related annotations; only 1 was entirely unvalidated; an additional 6 were validated for a non-human ortholog to the human protein without explicit mention of whether the prediction is expected to be valid for the human ortholog; and an additional 4 have what we would describe as “weak” human validation. The resulting validation rates are 18/19 (95%), 12/19 (63%), and 8/19 (42%). In the case of “weak” validation, it is possible that, rather than directly transferring the specified GO term, it may be more appropriate to transfer a less specific GO term higher in the GO hierarchy. Determining when this is the appropriate action is an area of future research.

- HDAC5 upregulates MEF2C; in turn, MEF2C is known to be missing during metastasis, the latter of which is necessary for ciliogenesis; conversely, inhibition of HDAC5 suppresses cyst formation that disrupts cilia formation^63^. HDAC5’s upregulation of MEF2C also causes malformed cilia which can be rescued by knockdown of MEF2C^64^; HDAC5 morphant Zebrafish exhibit shorter cili^65^.
- CAND1 is a centrosome protein known to regulate centrosome amplification; CAND1 knockdown in mice inhibits airway ciliogenesis^66^.
- RPL6 is a centrosomal marker among a selection of known or candidate centrosomal proteins [ref. ^67^, Figure 18.2].
- CUL7 Reduction in CUL7 expression is associated with defects in centrosome and cilia formation^68^.
- CFTR at the molecular level is involved in chloride transport, but loss of function of CFTR disrupts cilia in lung tissue, causing cystic fibrosis (CF); direct delivery of CFTR to the lung is an active research area in the fight against CF^69–71^.
- CDH1: there seems to be some controversy as to whether CDH1 does^72^, or does not^73^, affect cilia.
- HNRNPU: there is indirect evidence in a mouse model specifically designed to model human ciliopathy that HNRNPU interacts with SLP3^74^, a known cilia-active protein^75^.
- RPL18 (Ribosomal Protein L18) is one of 268 proteins identified in a rat cilia preparation [ref. ^76^, Table 1]; admittedly, the evidence here is weak as they make no further mention of RPL18.
- RNF2 is regulated by known BBS (cilial dysfunction) genes^77,78^.
- VCAM1 is expressed on the ciliary body of mouse retinal cells modeled to study human autoimmune disorders^79^.
- XPO1 aids ciliary Gli2 export in mice^80^.
- CUL5 knockdown weakly suppresses ciliogenesis in human epithelial cell cultures^81^.
- CCDC8, OBSL1, and CUL7 form a centrosomal complex^82^ in mice^83^ and cultured human cells^84^; this complex is implicated in *3M Syndrome* (same references, but also as studied in human HEK293T cells^85^).
- SOD1 mutations are of interest because they are associated with a minority of the familial version of the muscular disease ALS; it has been shown than SOD1 mutations inhibit ciliogenesis in motor neurons in mice^86^.
- RPS9 is known to be expressed in cells bearing motile cilia of model fish species^87^.
- C1ORF87 is found in high abundance in human airway cilia^88^.
- CNBP integrity of the primary cilium is necessary to induce CNBP in human cancer stem cells^89^.

We note that, of the GO term predictions in Table 5, 20 are Cellular Component (C), 11 are Biological Process (P), while none are Molecular Function (F). For this reason it would be misleading to label the results of this paper as “functional prediction”. The biggest contributing factor to the lack of functional predictions is likely the fact that, of the 285 cilia-related mouse annotations, 205 are Cellular Component, 71 are Biological Process, and only 9 are Molecular Function. Thus, there is simply a dearth of truly functional annotations of cilia-related mouse proteins from which to draw predictions. A second likely contributing factor is, again, the dearth of network data which likely allows proteins to be aligned *close* to their “proper” place in the network but not directly to their functional ortholog. We hypothesize that this latter issue will become less of a problem as more PPI data are accumulated.

#### Detailed validation of our single highest NAF prediction

The single highest NAF score was 82% between mouse protein Fancd2 and human protein TRIM25. Here we provide detailed literature-based validation of all GO terms present in mouse Fancd2 but not human TRIM25 in the Sept. 2018 GO release—cf. Table 6. Most are Biological Process GO terms, which according to recent CAFA^61^ benchmarks is the most difficult GO category to predict. Note that in this section, we no longer restrict ourselves to cilia-related GO terms, and we arbitrarily omit validation of GO terms predicting by the IMP evidence code, due to time constraints. Thus, the text below attempts validation only of GO terms predicted by evidence codes other than IMP, though IMP-based predictions are included in Table 6.

##### Biological Process GO:0048854 (brain morphogenesis)

Formation of the brain requires differentiation of stem cells into determined cell types. Autophagy plays an important role in stem cell differentiation, as it allows the cell to degrade obsolete organelles to become a more specialist cell ^90^. TRIM family proteins are emerging as important regulators of autophagy, and interact with a range of known autophagy proteins^91^. A number of autophagic genes, including Ambra1, are expressed in mouse embryos during neuronal differentiation^92^. Ambra1 has been shown to be a key modulator of neurogenesis^93^. Recently, it has been demonstrated that TRIM25 interacts with Ambra1 to up-regulate autophagy in mouse liver cells^94^. Whether TRIM25 interacts with Ambra1 similarly in neural cells is not known, but two of its close relatives have been shown to promote neural differentiation by different pathways: TRIM32^95^, and TRIM69^96^. TRIM25 has been shown to enhance transcriptional activity of the differentiator gene RAR*α* to a similar degree as TRIM32^95^, further implicating it in this pathway for promoting neural stem cell differentiation.

##### Biological Process GO:0097150 (neuronal stem cell population maintenance)

Understanding the functions of different TRIM proteins in this regard is an area of cutting-edge research, as discoveries that TRIM proteins have regulatory functions in neural development and maintenance have only recently been made^97^. As with stem cell differentiation, autophagy is an important process in stem cell maintenance^90^, and TRIM proteins have important roles in autophagy ^98,99^. Deficiencies in autophagy can result in neuro-degenerative disorders and premature aging^100^. TRIM25 is expressed and contributes to stem cell maintenance in mouse embryos^101^ by ensuring genomic stability following DNA replication^102^. A recent survey^97^ states that TRIM25’s function in stem cells appears to be the least well understood out of all TRIM family proteins, and makes no mention of a role for TRIM25 in neurological processes. The indirect evidence presented above, along with its high NAF score, suggests that TRIM25’s role in this area be further investigated.

##### Biological Process GO:0034599 (cellular response to oxidative stress (ROS))

Oxidative stress in cells is used as a signal of protein activity and function. Viral infection can lead to oxidative stress and degradation of viral proteins via proteasomes, and the TRIM25 ubiquitylation pathway^103^. Viral-origin proteins, when expressed in the cell, commonly generate reactive oxygen species. The RIG-1 pathway is known to respond to ROS to trigger cellular processes as part of the innate immune system^104^. Importantly, reactive oxygen species are also a known stimulus for activating autophagic processes^105^, providing an obvious potential link between this prediction and the autophagy ones discussed above.

##### Components GO:0000793 (condensed chromosome) and GO:0005634 (nucleus)

TRIMs have roles in cell cycle progression^106^. The cell cycle is composed of various different phases, one of which is mitosis (M phase). During mitosis, a number of changes occur within the cell, including the condensation of DNA into chromosomes (in prophase). While the review of Venuto and Merla^106^ does not acknowledge TRIM25 to have a specific role in prophase mitosis, the relatively uncharacterized status of TRIM25^97^ does not contradict our prediction. Finally, chromosome condensation occurs in the nucleus, so if TRIM25 is involved in condensing the chromosome, this additionally implies GO:0005634.

In sum, TRIM25 appears a poorly understood member of the TRIM family. Given the importance of E3 ubiquitin ligases in neurological development, disorders and degenerative conditions^107^ these predictions from PPI network alignment provide plausible directions for future research in the function of TRIM25.

### Comparison with other methods that use only network topology

At the time of writing, we are aware of only two methods in the literature that predict GO annotations using only network topology: SINaTRa^108^ and Mashup^109^; neither is based on network alignment.

Synthetic lethality (SL) refers to a pair of genes neither of which is alone essential to life, but death occurs if both are knocked out simultaneously. SINaTRa^108^ uses a vector of traditional (non-graphlet) local measures of network topology to quantify the neighborhood of a node, and then uses standard machine learning techniques to train an SL classifier on pairs of genes in one species, and then predict SL pairs in another species. While the authors attempt no other types of prediction other than SL, and they use data from just one year (2015), the closest approximation to our results are when they train on yeast (*S. cerevisiae*) and test on an “ablated” version of the fission yeast (*S. pombe*) network designed to mimic the edge density of the human network. In this test (their Figure S10), they achieved AUPRs between 0.43 and 0.60 [ref. ^108^, p. 9]. Their higher AUPRs may be attributable to their using more recent data (by 5 years).

Mashup^109^ uses network diffusion to construct a compact, low-dimensional vector of features for each node in a network. They then integrate the feature vectors extracted from many different types of networks from the same species to train an off-the-shelf machine learning algorithm to learn properties of interest, such as GO terms. Using the 2013 STRING database as input, they achieve AUPRs for prediction of human GO terms in the range of about 0.15 to 0.40 (their Figures 2 and 3), depending on details of their ranking. These numbers are comparable to ours (cf. Fig. 4 (bottom)).

## Discussion

In broad outline, our main results are:

1. Across many stochastically-generated inter-species network alignments with near-optimal^40^ topological scores, the frequency that a pair of proteins is aligned together correlates with, and has predictive value of, Resnik similarity.
2. NAF exposes Resnik similarity not only in the absence of significant sequence similarity, but exposes such similarity between non-sequence similar proteins that is just as strong as the Resnik similarity between sequence-similar proteins (cf. Fig. 3). This leads to the NAF-function Postulate (page 6).
3. While sequence comparison is obviously an accepted and valuable tool when predicting functional similarity, it is simply not applicable when no significant sequence similarity is detectable. Thus, sequence similarity is a sufficient but not necessary condition for inferring functional or semantic similarity (cf. Fig. 3).
4. To our knowledge, NAF is the first measure based solely on topology-driven network alignment to provide GO term predictions with success that is competitive with state-of-the-art methods, whether based on sequence, structure, or topology.

Though not depicted in any Figures, we also measured precision, recall, and AUPR of our 2010-based predictions (similar to Fig. 4 (bottom)) by validation against GO releases for every year from 2011 to 2019. We found that the number of validated predictions, sourced from 2010, increases significantly year-over-year, suggesting that many “non-validated” predictions may become validated at some future date. Also, though not discussed in the main text, Supplementary Fig. 1 demonstrates that the ability to detect and predict semantic similarity scales with degree and, more generally, edge density (see also our companion paper^40^). This leads us to predict that the following will occur as network data continue to accrue:

- Larger regions of the networks will become robustly alignable—i.e., NAF scores will increase, along with the number of protein pairs aligned with NAF above any fixed threshold.
- Topology-driven network alignments will be able to discover better topological agreement between networks, resulting in more GO term predictions, and with greater confidence. This hypothesis is corroborated by the much higher prediction accuracy of our literature validation of 2018-sourced predictions than those from 2010.
- In general, the biological relevance of topology-driven network alignments will increase dramatically.

Related to the above, it is important to emphasize that we are not claiming that the results expounded in this paper are of practical use—yet. The fundamental problem is dearth of PPI network data. Yeast and Human are by far the most complete species pair, and yet they do not produce the best predictions, possibly due to their great taxonomic distance. The mere fact that we had to run one hundred independent 1-h runs of SANA per species pair in order to tease out the weak signal attests to how weak that signal is at present. The signal is just too weak, and the CPU requirements too large, for the method to be practical on existing networks. We expect, however, that as PPI networks become more complete and less noisy, a much more clear signal will appear in network alignments, allowing topology-only network alignments to more efficiently extract predictions.

One may notice that the “good” values of NAF and other parameters of our algorithm varies widely between species. We believe this, again, is due to the wide disparity in network densities between species. This makes it fruitless to “tune” the parameters of our algorithm on one species pair and use those parameters on another pair. We also have not accounted for multiple hypothesis testing in any of the *p*-values herein.

Clearly, our goal is to make the best novel GO term predictions using today’s data. To do that, it is important to have an estimate for the confidence level of predictions made today when no validating data is available. We intend to explore the many relationships observed in this paper to get a better handle on how to assign a confidence to each prediction made. For example, we expect that as PPI data accumulate with time, predictions will be more precise and have higher confidence; this hypothesis is supported by the literature validation rates above applied to predictions using recent PPI data. However, the more recent the PPI data, the smaller the duration between the date of the prediction, and the date of validation; thus, validation rates will appear lower simply due to the lack of passage of time. Untangling these effects in order to produce predictions with a reliable confidence level is an obvious direction for future research.

## Methods

### Sequence similarity according to BLAST

For all analyses other than those in Table 5, we ran BLASTP locally with the default parameters to align all-to-all pairs of proteins between each species pair. Pairs of proteins were labeled as “having sequence similarity according to BLAST” if and only if BLASTP listed that pair anywhere in its output, otherwise not; the lowest observed bit score was 13.5, while E-values ranged from zero up to 1000. As a more sensitive test specifically applied to Table 5, we visited NCBI's PSI-BLAST page, and for each row we entered the accession code for the mouse protein and used the PSI-BLAST program choice. In all cases, many matches (dozens to hundreds) among human proteins were found with E-values ranging from 10 down to 1e−180, but in all cases we verified that none of those matches came from the protein in the Human column of Table 5.

### Formal definition of Pairwise Global Network Alignment

Let *G*_1_, *G*_2_ be two undirected graphs (i.e., networks), with node sets *V*_1_, *V*_2_ and edge sets *E*_1_, *E*_2_. Let *n*_*i*_ = ∣*V*_*i*_∣, *i* = 1, 2 be the number of nodes in the networks, and *m*_*i*_ = ∣*E*_*i*_∣, *i* = 1, 2 be the number of edges in each. Without loss of generality, assume *n*_1_ ≤ *n*_2_. We define a *global* network alignment *a* as a 1-to-1 function *a*: *V*_1_ → *V*_2_ that maps each node in *G*_1_ to some node in *G*_2_. (While the 1-to-1 requirement does not handle all biologically relevant cases, it is a widely adopted assumption; however, SANA’s randomness effectively eliminates this restriction.) Figure 1 provides a simple schematic example of such a network alignment.

### GO term prediction and automatic validation

The following description applies only to automatic prediction and validation, not to manually literature-curated validations.

Assume we have two species *s*_1_, *s*_2_. Our goal is to use the PPI networks and GO annotations of both species available as of date *t* to predict the existence of novel GO annotations not available at time *t*, and validate these predictions using GO terms available at some later date $t^{\prime} \,>\, t$. Without loss of generality assume we are making predictions in the direction *s*_1_ → *s*_2_, i.e., using GO annotations of proteins in *s*_1_ to predict GO annotations of proteins in *s*_2_. We refer to *s*_1_ as the *source* species, and *s*_2_ as the target species. In our case we are making predictions using networks and annotations available at *t* = April 2010 (BioGRID 3.0.64 and GO release 2010-04, both available in April 2010), and validating those predictions using annotations available from the GO release at $t^{\prime} =$ April 2020. The GO databases were retrieved from the EMBL-EBI UNIPROT historical GO database, which specifically focuses on protein (as opposed to gene) function.

Assume that on date *t*, species *s*_1_, *s*_2_ have PPI networks, *G*_1_, *G*_2_ with node sets *V*_1_, *V*_2_, and let *n*_1_ = ∣*V*_1_∣, *n*_2_ = ∣*V*_2_∣. Node sets consist of ${V}_{1}={\{{p}_{i}\}}_{i = 1}^{{n}_{1}},$ and ${V}_{2}={\{{q}_{j}\}}_{j = 1}^{{n}_{2}}$. For simplicity we will drop the node subscripts and refer to *p* ∈ *V*_1_ and *q* ∈ *V*_2_. Assume that on date *t*, *p* is annotated with GO terms *γ*_*p*_, and *q* is annotated with GO terms *γ*_*q*_. We will use the same letters for all entities at the later date $t^{\prime}$, but with a prime added: for example ${G}_{1}^{\prime}$ refers to the PPI network of *s*_1_ at time $t^{\prime}$, $p^{\prime}$ refers to a protein in ${V}_{1}^{\prime}$, and ${\gamma }_{p^{\prime} }$ refers to the set of annotations to $p^{\prime}$ at time $t^{\prime}$. Note that $p^{\prime}$ and *p* are the same protein, but there exist proteins that may only exist in one of the two PPI networks, or one of the two GO annotation databases; thus, *p* may exist in the PPI network at time *t* but have no GO annotations at that time, or vice versa. (Note we do not include proteins with degree zero in our PPI networks, since they possess no useful topological information.)

We say that the association of GO term *g* with protein $q^{\prime}$ of the target species *s*_2_ at time $t^{\prime}$, sourced from any protein *p* in *s*_1_ at time *t*, is predictable in principle if both of the following are true:

- *q* ∈ *V*_2_—i.e., the protein exists in the earlier PPI network of the target species *s*_2_. This is because *q* acquires annotations from proteins in the source species by being aligned to them at time *t*; *q* cannot be aligned if it does not exist in *G*_2_.
- ∃ *p* ∈ *V*_1_ such that *g* ∈ *γ*_*p*_—i.e., at least one protein from source species *s*_1_ is annotated with *g* at the earlier time. (Otherwise there is no place from which to source *g* as a prediction for $q^{\prime}$.)

We define *P*_12_ as the set of all such predictable in principle annotations from species 1 to species 2; this set is derivable from information known only at the earlier time. Note, however, that its size is huge, being the product of the number of nodes in *s*_2_ at time *t* and the number of distinct GO terms annotating *s*_1_ at time *t*.

Note that, although *q* needs to be in the earlier network *V*_2_, we do not demand that it exists in either of the GO term databases; those that exist in the later but not the earlier GO database, and for which we can make predictions, count as completely unannotated proteins at the earlier time, for which we may be able to make, and validate, predictions; those that also fail to exist in the later GO database may have predictions that are not yet, but may ultimately become, validated. Finally, we say that a predicted annotation $(v^{\prime} ,g)$ is validatable if $g\in {\gamma }_{v^{\prime} }$—that is, *g* annotates $q^{\prime}$ in the later GO database.

To measure recall, we need a maximal set of “ground-truth” annotations at the later date. The most obvious candidate “ground truth” is all GO annotations across all proteins in the target species at the later date, which we call ${{{\Gamma }}}_{2}^{\prime}$. However, there are likely to exist annotations $(v^{\prime} ,g)\in {{{\Gamma }}}_{2}^{\prime}$ that are not predictable in principle as defined above, either because *g* annotated no proteins in *V*_1_, or because *q* had no known interactions at time *t* and thus did not exist in *G*_2_. Thus, we define our maximal “ground truth” set as ${P}_{12}\cap {{{\Gamma }}}_{2}^{\prime}$, and the number of elements in that set becomes the denominator in our computation of Recall.

We use AUPR rather than ROC curves because the data are extremely unbalanced: in particular, $| {P}_{12}| \gg | {{{\Gamma }}}_{2}^{\prime}|$, directly informing us that the negative set is much larger than the positive one. For example, in April 2010, the human BioGRID PPI network had 8192 nodes, and the other species listed above all had 3000–10,000 GO terms, so ∣*P*_12_∣ is in the tens of millions, but the number of validating annotations for human in 2020 is <20,000, making the negative set about 1000 times larger than the positive one.

We make every attempt to eliminate any prediction that could have been made or validated using sequence analysis. In particular, we

- eliminate any protein pairs (*p*, *q*), regardless of NAF, which have sequence similarity according to BLAST (bit score threshold of 13), or those with known (even distant) orthology according to NCBI Homologene^110^, InParanoid 8^56^, or the 2019 release of EggNog 5^57^;
- eliminate any GO terms of *p* possessing evidence codes from Table 1 (bottom), even if they also possess non-sequence-based evidence.
- discard any “predicted” annotations that were already known at time *t* between *q* and GO terms with any evidence code (including those in Table 1 (bottom));
- discard any predicted annotations for which sequence evidence had been produced by time $t^{\prime}$.

We are left with predictions of GO terms annotating $q^{\prime}$ that were entirely unknown at time *t*, that came from GO annotations of *p* at time *t* that did not possess any sequence-based evidence, and that still lacked sequence-based evidence as of time $t^{\prime}$, even when including orthology based on the best homology methods of time $t^{\prime}$. Note that for consistency, when we remove any predictions coming from a pair of proteins (*p*, *q*) using the above criteria, we also remove the predictions from *P*_12_ unless the same prediction can be sourced from another protein $\hat{p}$ in *s*_1_ that is not eliminated based on the above criteria. (That is, we eliminate it from both the numerators and denominators of precision and recall.)

Using these criteria, we feel confident that the majority of (possibly all) predictions discussed in this paper were unattainable by any other means using data or methods available as of *t* = April 2010, and additionally had still not been discovered by any sequence or homology-based method available as of $t^{\prime}$ = April 2020.

## Supplementary information

Supplementary Information

## Data Availability

BioGRID networks are available from BioGRID.org; GO term releases are available at GeneOntology.org. The output alignments, including alignment frequency, Resnik score, and paired proteins used to generate all Figures in the manuscript are available for the EC measures in the paper at http://sana.ics.uci.edu/SANA-predicts-GO-terms/Topo-Function-2019-alignments-EC.7z, and for a longer list of objectives (many of which were used in our companion paper^40^) at http://sana.ics.uci.edu/SANA-predicts-GO-terms/Topo-Function-2019-alignments-all.7z.

## Code availability

Source code for SANA is available at our GitHub repository; the Resnik Python script, which uses the FastSemSim library^44^, is part of the SANA repo.
