# Supplementary material for: SANA: cross-species prediction of Gene Ontology GO annotations via topological network alignment
Source: NPJ Syst Biol Appl. 2022 Jul 20;8:25. doi: 10.1038/s41540-022-00232-x (PMC9300714; doi:10.1038/s41540-022-00232-x)
Supplement: Supplementary file 1 — Supplementary Information [file 41540_2022_232_MOESM1_ESM.pdf]

Supplementary Info

SANA: Cross-Species Prediction of Gene Ontology GO Annotations via Topological Network Alignment

Siyue Wang, Giles R. S. Atkinson, and Wayne B. Hayes (whayes@uci.edu)
Department of Computer Science, University of California, Irvine, CA 92697-3435, USA

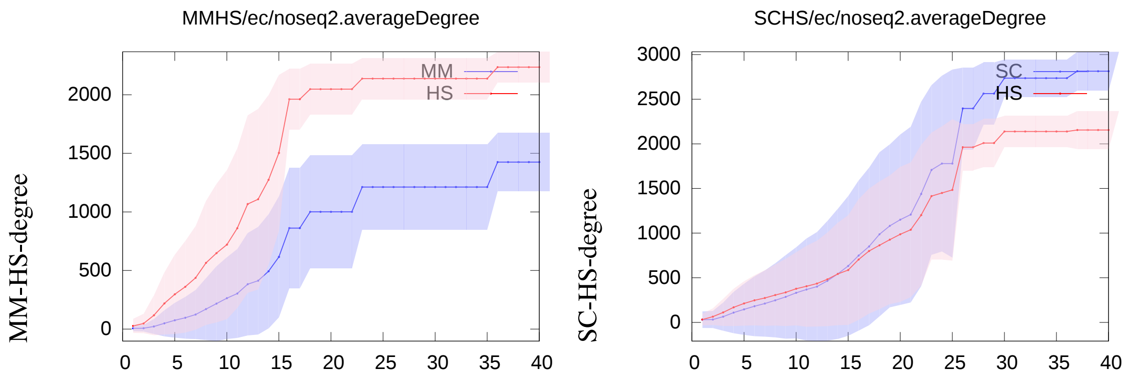

Figure 1. Mean and standard deviation of degree of aligned protein pairs, as a function of NAF (%), for the same parameters and species as Main Manuscript Figure 2. In every plot, red is human, and blue is the other species. We observe that nodes with higher degree in the original networks tend to be more frequently aligned. The depicted mouse-human Pearson correlation is 0.92, while yeast's is 0.65; both p-values are below 10^{-300}.

The effect of edge density and degree

We expect that edge density strongly affects alignment robustness, simply because the more edges we have, the more topological information we have to align similar regions(1). We can quantify this at the node level by observing how node degree correlates with NAF. Figure 1 depicts the relationship between node degree and NAF. We can clearly see that higher degree nodes are more reliably aligned than lower degree ones.

One of the referees pointed out that since both Resnik and mean degree correlate with NAF, there is the possibility that NAF simply aligns node pairs with high degree; since both degree and GO annotation level are correlated with "popularity" of proteins in the research literature (2), increased popularity would result in both higher node degree and more GO annotations, potentially contributing to the Resnik-NAF correlations observed in Figures (main manuscript) 2 and 2. This hypothesis can be tested in at least three ways. First, assume we list *all* pairs of proteins $p \in G_1, q \in G_2$. Then sort the list of pairs by degree—for example sorted by the arithmetic or geometric mean degree of the two, or by the minimum or maximum degree of the two. We have computed the mean Resnik similarity across all mouse-human protein pairs, and indeed, we find an *enormously* powerful correlation between degree and Resnik: degree-1 node pairs have mean Resnik scores of about 0.5, which rises dramatically up to a Resnik value of about 3.5 at degree 30. Unfortunately, this trend halts abruptly at that point: there are over 600,000 pairs of mouse-human proteins in which *both* have degrees above 30, which is higher by many orders of magnitude than the number of high-Resnik pairs in Figures (main manuscript) 2 and 2; however, their mean Resnik score remains constant at about 3.5 for all degree thresholds above 30. Thus, degree alone cannot be responsible for the results of Figures (main manuscript) 2 and 2. A second argument against the Resnik-degree hypothesis is by our companion paper(1), which clearly shows that our alignments recover orthologous protein pairs—essentially the strongest definition of a "correctly" aligned pair between species—at a rate *far* higher than random, which again eliminates high degree as the sole cause of good alignments (though of course they *enable* good alignments by providing more information). Third, many of the correctly recovered orthologs in our companion paper(1) do not have particularly high node degree: there were 16 orthologs in which the mouse protein has a degree less than the median, 10 for which the human one does, and 7 for which both do, for a grand total of 19 instances of correctly aligned orthologs with degree below the global median. Since a randomly chosen protein has a 50% probability of having degree below the median, these low-degree ortholog alignments have a collective probability of 2^{-19} (one in half a million). Again we conclude that higher degree *enables* good alignments but does not, in and of itself, cause them.

species	max($\overline{D}$)	EC			S^3			Importance			graphlet			lgraal		
		NAF	nodes	$\overline{D}$	NAF	nodes	$\overline{D}$	NAF	nodes	$\overline{D}$	NAF	nodes	$\overline{D}$	NAF	nodes	$\overline{D}$
SC-DM	28.99	5	874	28.84	5	870	28.99	5	774	28.16	4	21135	0.14	4	20466	0.15
SC-HS	14.10	4	1291	12.91	4	1186	14.10	4	1235	12.84	5	18504	0.28	4	19593	0.26
MM-DM	12.70	7	101	10.93	6	89	11.26	8	46	12.70	19	453	0.44	11	757	0.38
SP-SC	12.58	3	2116	7.96	6	67	9.64	9	24	12.58	80	287	0.08	4	3525	0.20
SP-DM	12.23	8	108	11.57	8	60	12.23	8	63	12.19	7	2843	0.30	12	2171	0.14
CE-SC	11.38	4	1093	11.38	4	348	8.30	5	219	10.09	90	430	0.04	5	11027	0.04
AT-HS	10.25	4	879	8.56	4	501	8.89	5	340	10.25	19	1872	0.52	13	2631	0.32
AT-DM	10.23	4	831	7.85	3	1421	6.63	9	69	10.23	30	1281	0.32	12	2922	0.18
CE-HS	9.77	4	808	9.04	5	272	9.77	5	312	9.26	30	2147	0.27	13	4438	0.28
MM-CE	9.48	6	313	6.93	10	112	9.45	9	142	9.48	12	649	0.68	17	513	0.75
MM-SP	9.33	7	246	9.33	9	94	7.98	8	117	8.34	13	638	0.53	6	1554	0.50
SP-AT	9.22	7	1022	6.91	6	978	8.01	7	704	9.22	13	1920	0.85	12	2096	0.54
AT-SC	9.15	4	896	9.15	4	565	8.00	4	514	8.21	30	1027	0.08	4	6094	0.14
DM-HS	9.11	3	1696	5.33	3	1548	5.00	4	690	9.11	8	21033	0.23	5	27210	0.36
AT-CE	8.32	6	786	6.79	7	513	7.52	11	194	8.32	12	2773	0.54	7	4988	0.39
MM-SC	7.73	3	388	2.84	3	337	5.49	5	60	7.73	90	47	0.09	40	180	0.06
SP-CE	7.52	7	438	7.32	12	84	7.52	9	137	7.30	13	1953	0.49	4	4075	0.37
CE-DM	6.23	4	538	5.80	4	374	5.14	5	175	6.23	9	5962	0.26	15	3811	0.31
SP-HS	5.62	3	1991	5.38	4	188	5.31	7	47	5.62	15	1634	0.41	15	1700	0.23
MM-HS	5.02	3	533	3.67	4	139	5.02	4	157	4.47	13	687	0.93	14	609	0.51
RN-CE	4.78	6	71	3.77	13	23	4.78	17	15	4.67	20	146	0.81	12	245	0.69
MM-AT	4.47	4	927	4.47	20	19	4.21	5	497	3.81	8	1137	1.18	11	763	0.60
RN-SP	4.33	7	24	4.33	4	297	2.34	5	145	2.07	30	118	0.66	40	87	0.55
RN-DM	3.93	4	61	3.93	3	152	2.54	4	63	2.83	17	203	0.60	13	245	0.57
RN-AT	3.12	4	191	3.12	3	663	1.80	3	665	1.96	30	104	0.90	11	273	0.70
RN-MM	3.00	6	471	3.00	9	114	2.32	8	188	2.41	14	6	1.33	16	206	0.87
RN-SC	1.32	2	1116	1.32	2	745	0.99	2	714	1.02	100	26	0.08	50	56	0.07
RN-HS	1.15	2	936	1.14	2	661	1.13	3	96	1.15	30	122	0.85	12	258	0.53

Table 1. Mean degree of the *Common Connected Subgraph* (CCS, cf. purple edges in Main Manuscript Figure 1) induced on the aligned node pairs with NAF above the threshold in the NAF column. For each species pair and measure, we list only the set of nodes with the highest mean degree and the value of NAF that gave it. The table includes all $\binom{8}{2}$ species pairs from Main Manuscript Table 1(top), and is sorted by the first column (the maximum mean degree across all 5 measures). Note that RN-HS (rat-human) has the lowest mean degree across all 28 pairs.

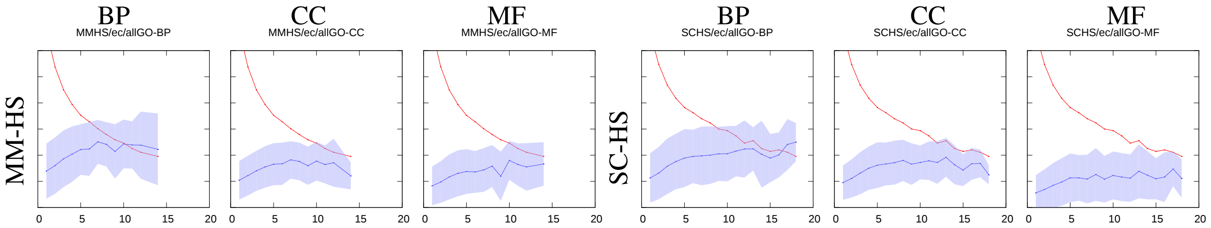

Figure 2. Splitting GO into Biological Process, Cellular Component, and Molecular Function: We plot mean (blue line) and standard deviation (blue shade) of Resnik scores vs. Network Alignment Frequency (NAF%), split into the three GO Categories. The left three panels depict mouse-human, and the right three are yeast-human. All alignments are driven to optimize EC. We use the same axes as Main Manuscript Figure 2. To reduce clutter, we only plot the version corresponding to Main Manuscript Figure 2's second sub-plot (all aligned pairs, all GO terms). The relationships all have Pearson correlations in the range $[0.05, 0.10]$ and p-values below 10^{-30}.

Sequence-similar pairs have more *non*-sequence evidence

Supplementary Figure 3 provides a more in-depth analysis of the difference observed between the "allGO" vs. "NOSEQ" parts of Main Manuscript Figure 2. Part (a) plots the curves of mouse-human mean NetGO-weighted mean Resnik scores as a function of frequency, with core sizes and standard deviations removed for clarity. We observe more clearly the stark difference between scores of pairs with and without sequence. The most obvious possible explanations are (i) pairs with sequence similarity are genuinely closer in Resnik similarity than those without, or (ii) the two sets have comparable Resnik similarity but the computed Resnik score is higher for those with sequence similarity simply because there is more information available for such pairs. Part (b) of the figure tries to resolve the ambiguity by removing *all* sequence-based GO terms, including those that have been human curated—because human curation does not change the fact that nobody would even have *looked* at the possibility of functional or semantic similarity unless an automated method first uses sequence to suggest the possibility. Surprisingly, even after removing all sequence-based GO terms, sequence-similar pairs *still* have a slight edge in mean Resnik similarity. In part (c), we find a potential explanation: we find that pairs of proteins that exhibit sequence similarity have, on average, two more *experimental* GO terms than pairs that do not have sequence similarity. Observe from Figure 4 that the mean *pairwise* Resnik score tends to increase with number of annotations; we hypothesize that perhaps there is a publication bias in GO terms similar to the publication bias that results in PPI network edges being biased towards pairs of highly studied proteins(3; 5): perhaps protein pairs that share sequence similarity tend to have more experimental tests for functionality than those that do not. In any case, we have at the very least demonstrated that the difference between "allGO" and "NOSEQ" *vastly* diminished if we remove sequence-related GO terms. Though not entirely satisfactory, we think it is sufficient grounds to continue to our next point, which is Part (d) of Figure 3, which is to point out that there are vastly more non-sequence-similar pairs at a given NAF alignment frequency than there are sequence-similar pairs. Circling back to part (a), recall that the separation of the green and purple curves was done after-the-fact—the *only* independent variable was network alignment frequency. Thus, since FastSemSim cannot use sequence similarity that doesn't exist, and if we trust the high Resnik similarity computed for those pairs that *do* share sequence similarity, it follows that all pairs at a given network alignment frequency should be drawn from the same distribution of Resnik similarity, and so NAF has the potential to fundamentally alter the landscape of protein functional predictions.

References

1. Wang, S. *et al.* On the current failure—but bright future—of topology-driven biological network alignment. *Advances in Protein Chemistry and Structural Biology (accepted; preprint https://doi.org/10.48550/arXiv.2204.11999)* (2022).

2. Luck, K., Sheynkman, G. M., Zhang, I. & Vidal, M. Proteome-scale human interactomics. *Trends Biochem. Sci.* (2017).

3. Rolland, T. *et al.* A proteome-scale map of the human interactome network. *Cell* **159**, 1212–1226 (2014).

4. Luck, K. *et al.* A reference map of the human protein interactome. *bioRxiv* 605451 (2019).

5. Walhout, A. J. & Vidal, M. Protein interaction maps for model organisms. *Nat Rev Mol Cell Biol* **2**, 55–62 (2001).

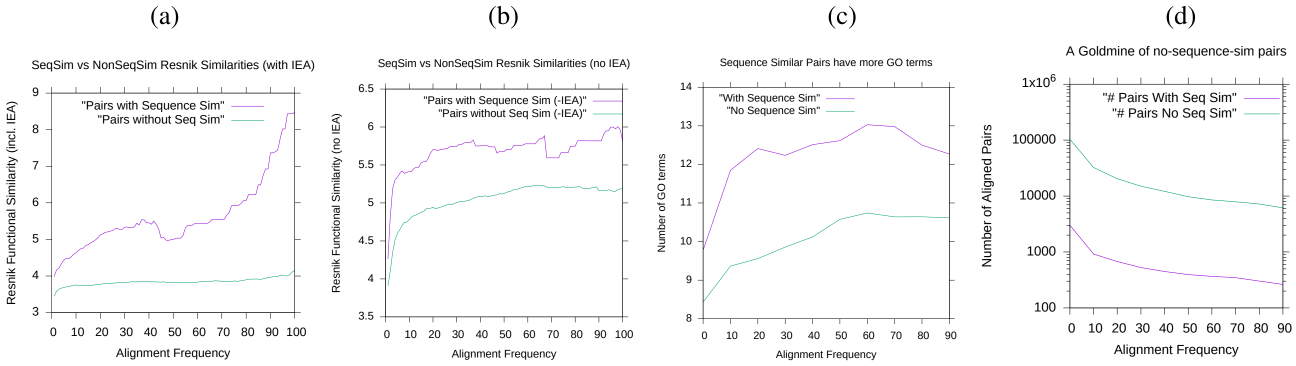

Figure 3. The effect of BLAST scores and sequence-based GO terms A closer study of the allGO vs. NOSEQ aspect of Main Manuscript Figure 2. We use purple curves to depict aligned pairs with *any* sequence similarity, and green for those that do not, both according to BLAST. **(a)**: We re-plot on the same graph the mean mouse-human Resnik scores, with core sizes and standard deviations removed for clarity. Clearly the scores are far better for pairs with sequence similarity. **(b): Removing sequence from the evaluation entirely:** Same as (a) but we remove *all* GO evidence codes based on sequence—including human curated ones: IEA, ISS, ISO, ISA, ISM, IGC, and RCA were all removed. Though diminished significantly, the effect persists. **(c): Mean number of experimental GO terms for pairs with and without sequence similarity** We find that pairs with sequence similarity have more *experimental* GO terms. This is likely a social / "popularity" bias similar to the known bias in PPI edge selection towards "interesting" proteins(2; 3; 4). For example, PIs may intentionally repeat the same experiment on known ortholog for confirmation/validation, or observe someone else's experiment on protein p in species X, and attempt to repeat it on a ortholog of p in species Y. **(d): Non-sequence-similar pairs dominate:** At a given network alignment Frequency, NAF produces far more protein pairs without sequence similarity than with. Finally, we note that this Figure is not in conflict with Main Manuscript Figure 3 since, in the latter, we have *forced* a comparison betwneen protein pairs with equal annotation levels (ie., the disparity in part (c) of this Figure has been removed).

rank	F^*	NAF	pair	M	Category	$\lvert P_{12} \cap \Gamma'_2 \rvert$	pred	valid	Precision
1	0.413	8%	DM-HS	S^3	Func	3510	3207	1386	43.2%
2	0.410	8%	DM-HS	Import.	Func	3510	3248	1387	42.7%
3	0.403	2%	CE-HS	S^3	Func	4572	3593	1644	45.8%
4	0.401	3%	CE-HS	Import.	Func	2743	2351	1021	43.4%
5	0.400	7%	DM-HS	EC	Func	3510	3231	1348	41.7%
6	0.400	16%	SC-HS	S^3	Func	3458	3367	1364	40.5%
7	0.398	16%	SC-HS	Import.	Func	3458	3479	1382	39.7%
8	0.396	16%	SP-AT	Import.	Func	480	645	223	34.6%
9	0.384	16%	SP-AT	S^3	Func	480	656	218	33.2%
10	0.336	16%	SC-HS	EC	Func	3458	2752	1043	37.9%
11	0.336	16%	SP-AT	EC	Func	480	699	198	28.3%
12	0.330	16%	MM-AT	Import.	Func	558	539	181	33.6%
13	0.321	16%	MM-AT	S^3	Func	558	538	176	32.7%
14	0.278	3%	AT-HS	S^3	Func	5196	4455	1341	30.1%
15	0.276	3%	AT-HS	Import.	Func	5196	4404	1326	30.1%
16	0.276	2%	CE-HS	EC	Func	3041	2691	791	29.4%
17	0.266	3%	SP-HS	Import.	Func	2872	3358	830	24.7%
18	0.264	3%	SP-HS	S^3	Func	2872	3438	833	24.2%
19	0.234	32%	SP-AT	Import.	Comp	1718	3439	604	17.6%
20	0.226	32%	SP-AT	S^3	Comp	1718	3427	582	17.0%

Table 2. 2010-based predictions, by GO Category. Similar to Main Manuscript Table 4 but for Categories.

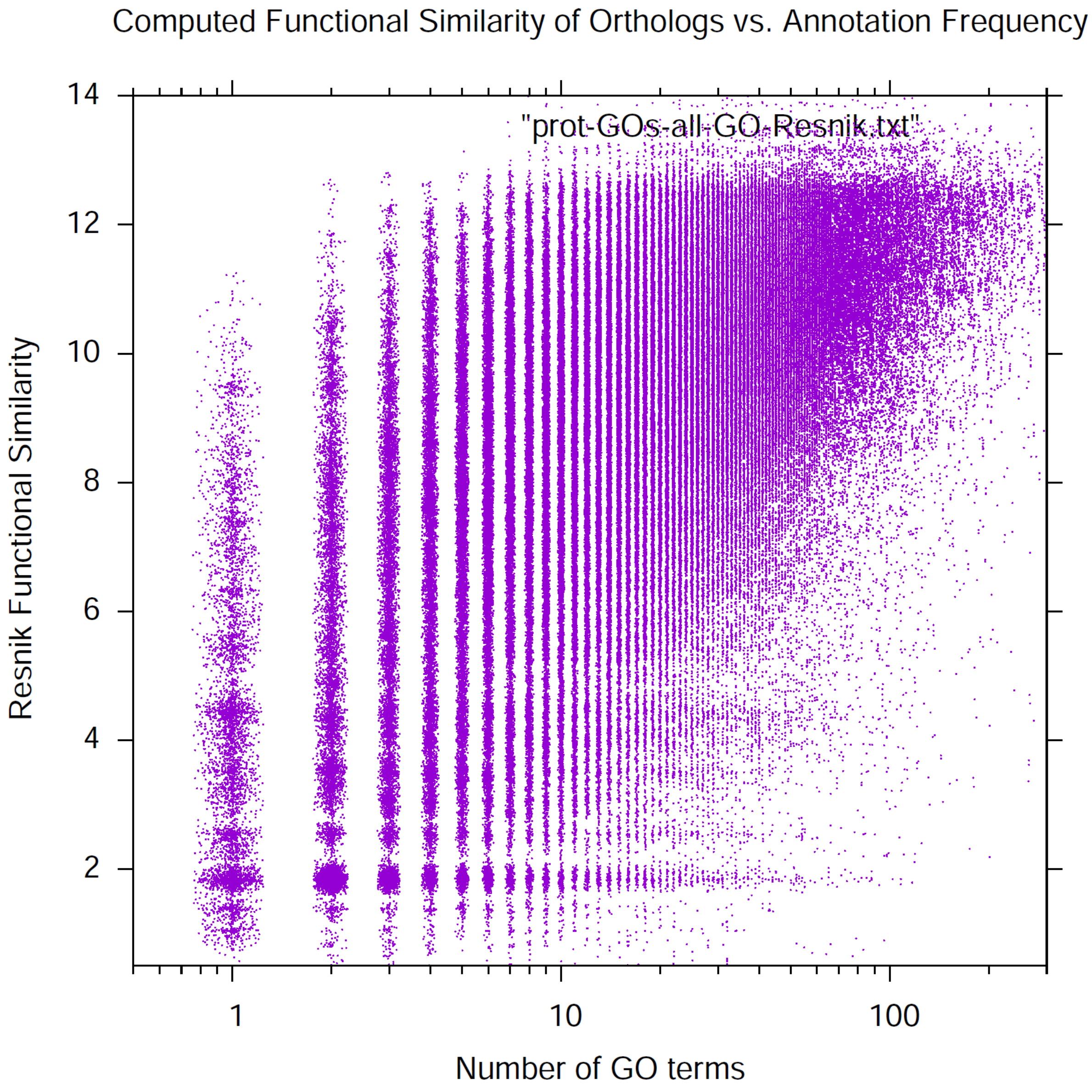

Figure 4. **Computed Resnik similarity between orthologs, as a function of GO annotation count** Each dot represents a pair of orthologous proteins between pairs of BioGRID species from Main Manuscript Table 1(top). The vertical axis is the computed Resnik semantic similarity between a pair of orthologs, while the horizontal axis is the GO term count of whichever of the pair has fewer GO annotations. Since they are orthologs, we expect them usually to have high Resnik similarity. We see this is true as long as both proteins are well-annotated, but fails when at least one is poorly annotated. (A small amount of random "jitter" has been added in both the horizontal and vertical directions to more clearly depict the density of points across the surface.)

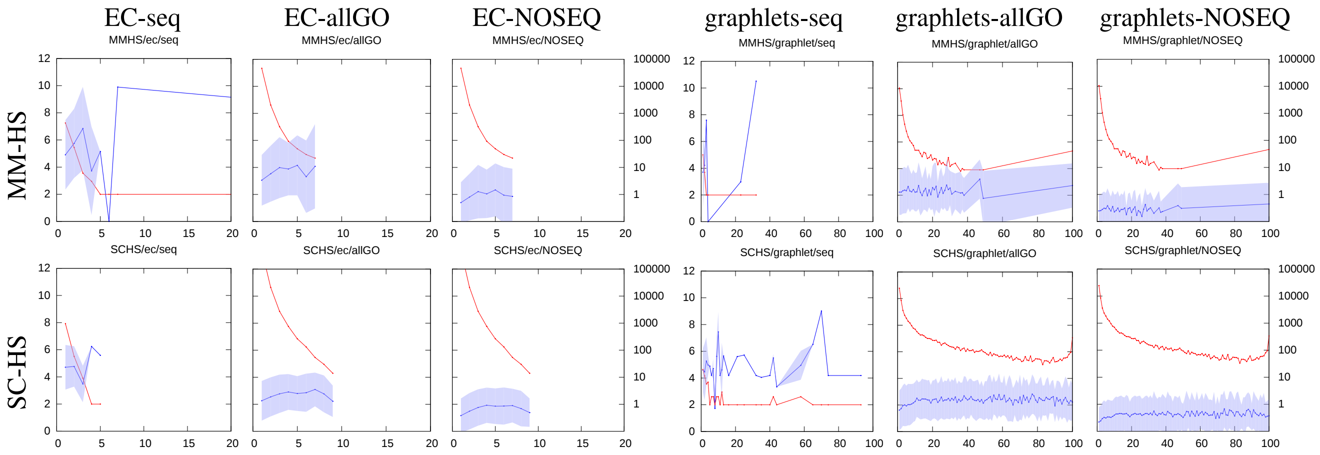

Figure 5. **Resnik-vs.NAF between mouse-human (top) and yeast-human (bottom) in the year 2010** Similar to Main Manuscript Figure 2 except using BioGRID 3.0.64 (released Apr. 23, 2010) and GO terms released the same month).
